# Dynamic changes of the immune microenvironment in ovarian cancer following neoadjuvant chemotherapy

**DOI:** 10.1038/s41420-026-03070-6

**Published:** 2026-03-23

**Authors:** Mingjie Wu, Fei Lv, Yi Jin, Shuai Wang, Lijun Meng, Jinyi Tong, Ruoyao Zou

**Affiliations:** 1 Department of Emergency Trauma Center, The First Affiliated Hospital of Zhejiang University School of Medicine, Hangzhou, China; 2 The Fourth School of Clinical Medicine, Hangzhou First People’s Hospital, Zhejiang Chinese Medical University, Hangzhou, China; 3 Department of Obstetrics and Gynecology, Affiliated Hangzhou First People’s Hospital, School of Medicine, Westlake University, Hangzhou, China; 4 Department of Gynecologic Oncology, Obstetrics & Gynecology Hospital of Fudan University, Shanghai, China

**Keywords:** Ovarian cancer, Cell growth

## Abstract

Standard treatment for advanced ovarian cancer involves initial surgery followed by platinum-based chemotherapy. Although most patients are sensitive, most relapses occur at a later stage, highlighting the urgent need to understand the tumour microenvironment following neoadjuvant chemotherapy (NACT). To explore the mechanisms underlying chemotherapy resistance, we analysed published single-cell RNA sequencing (scRNA-seq) data from patients with high-grade serous ovarian cancer and performed several in vitro and in vivo experiments to investigate the role of prostaglandins in immunosuppressive microenvironment formation. Prostaglandin-mediated immunosuppressive microenvironment formation was a critical contributor to chemotherapy resistance following cisplatin treatment. Mechanistically, cisplatin-treated ovarian cancer cells induced the formation of myeloid-derived suppressor cells (MDSCs), which inhibited the cytotoxicity of CD8^+^T cells. Combination therapy with cisplatin and a prostaglandin-specific inhibitor restored CD8^+^T cell function and significantly improved the therapeutic efficacy compared with cisplatin monotherapy. Targeting prostaglandins may be a promising therapeutic strategy for overcoming chemotherapy resistance in ovarian cancer.

## Introduction

Ovarian cancer remains one of the most lethal gynaecological malignancies worldwide, ranking as the eighth leading cause of cancer-related deaths among women, with a mortality rate exceeding 70% in advanced-stage cases [1]. Notably, 70–80% of patients are diagnosed at advanced stages (clinical stage III/IV), contributing to poor 5-year survival rates (~30–50% overall) [2, 3]. The standard treatment regimen includes cytoreductive surgery combined with platinum-based chemotherapy (carboplatin and paclitaxel). For advanced cases, neoadjuvant chemotherapy (NACT) followed by interval cytoreductive surgery (ICS) is increasingly being adopted to reduce the tumour burden and improve surgical outcomes [4]. Targeted therapies, such as PARP inhibitors (e.g. niraparib and olaparib), have revolutionised maintenance treatment, particularly for patients with *BRCA* mutations or homologous recombination deficiency (HRD). The PRIMA trial demonstrated significant progression-free survival (PFS) benefits with niraparib [5]. Bevacizumab, an anti-angiogenic agent, has also been integrated into first-line and recurrent settings to delay progression [6]. Even after successful initial treatment, 60–70% of patients relapse within 2–3 years, with over 80% of advanced patients developing platinum resistance, limiting therapeutic options [7, 8]. Therefore, more effective therapeutic methods should be developed to treat recurrences.

Ovarian cancer is characterised by a complex and immunosuppressive tumour microenvironment (TME) that dynamically evolves during treatment. The interplay among cancer cells, immune cells, and therapeutic interventions significantly influences disease progression and therapeutic outcomes [9]. Prior to treatment, the TME of ovarian cancer is predominantly immunosuppressive, characterised by high infiltration of tumour-associated macrophages (TAMs), particularly TREM2^+^ or CD163^+^ subtypes [10, 11]. It also lacks cytotoxic CD8^+^ T cells and has an elevated CD4^+^/CD8^+^ T-cell ratio, indicating impaired effector T-cell function [12], along with elevated Tregs, which suppress antitumour immunity by inhibiting CD8^+^ T-cell activity [13]. However, NACT transiently reshapes the TME, manifested by reduced neutrophil and monocyte counts, while temporarily boosting lymphocyte proliferation, creating a ‘window period’ of enhanced Th1 cell-mediated immunity that may synergise with immunotherapy [14]. Moreover, platinum-based chemotherapy reduces the proportion of Tregs, which correlates with a decreased tumour burden [13, 14]. However, other studies have indicated that immune suppression, including TAM infiltration, is not altered by neoadjuvant therapy [15]. This evidence suggests the complexity of the immune microenvironment in ovarian cancer and the high heterogeneity observed among patients, underscoring the necessity for mechanistic investigations into the dynamic alterations of the immune landscape following neoadjuvant chemotherapy and for the identification of potential therapeutic targets. We analysed scRNA-seq data from human ovarian cancer samples (data analysis workflow in Fig. S1A) and performed in vitro and in vivo experiments to determine whether the combined application of platinum and prostaglandin-specific inhibitors enhances the cytotoxicity of CD8^+^ T cells and significantly improves the efficacy rate compared to platinum monotherapy.

## Results

### Differential abundance testing reveals NACT-relevant non-tumour single cells and intrinsic signalling events

**Fig. 1:**
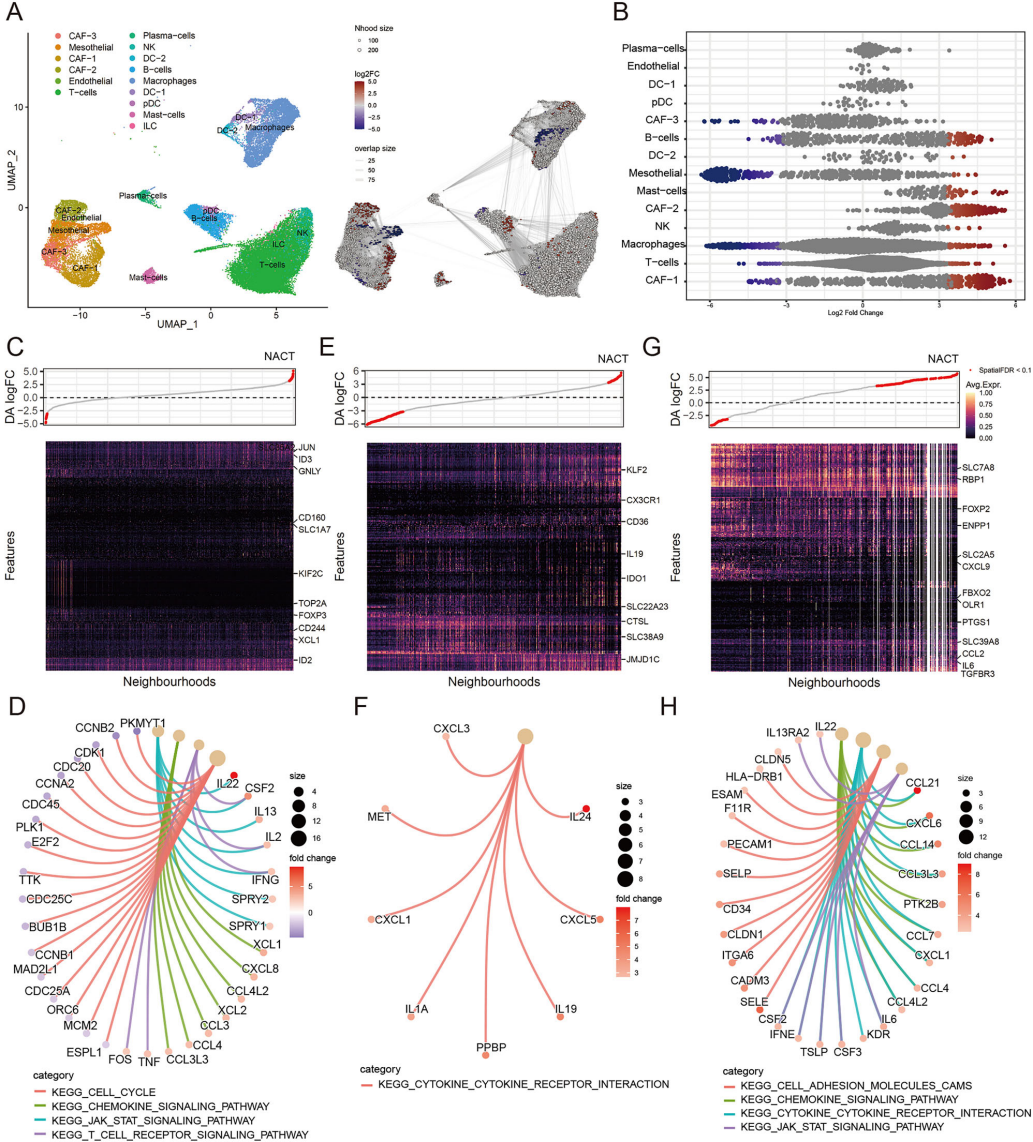
Transcriptional remodelling of the tumour microenvironment following NACT. **A** Uniform manifold approximation and projection (UMAP) embedding of transcriptomic data depicts 15 clusters of cell populations within the tumour microenvironment, annotated by cell type. The graph on the right indicates the assignment of neighbourhoods to discrete categories using Louvain clustering. **B** Differential abundance (DA) analysis of major cell types after NACT. Each dot represents a neighbourhood, coloured by log_2_FC (FC fold change) and grouped by annotated cell types. Differential abundance testing was performed by assigning cells to partially overlapping neighbourhoods using the k-nearest neighbour graph method. This analysis identifies perturbations obscured by the distribution of cells into clusters. **C** DA heat maps showing feature-level changes across cell neighbourhoods post-NACT in T cells. Differentially expressed genes are indicated on the right side of the figure. **D** Pathway enrichment analysis of the differentially abundant post-NACT features. Pathways were visualised using fold change (colour scale) and gene count (node size), highlighting enrichment in the cell cycle, cytokine signalling, adhesion molecules, and immune interactions. **E** DA heat maps showing feature-level changes across cell neighbourhoods after NACT in macrophages. Differentially expressed genes are indicated on the right side. **F** Pathway enrichment analysis of differentially abundant post-NACT features. Pathways were visualised using fold change (colour scale) and gene count (node size), highlighting the enrichment in cytokine–cytokine receptor interactions. **G** DA heat maps showing feature-level changes across cell neighbourhoods after NACT in fibroblasts. Differentially expressed genes are indicated on the right side. **H** Pathway enrichment analysis of differentially abundant post-NACT features. Pathways were visualised using fold change (colour scale) and gene count (node size), highlighting enrichment in chemokine signalling, JAK-STAT (janus kinase- signal transducer and activator of transcription) signalling, and cytokine–cytokine receptor interactions.

scRNA-seq data from human high-grade serous ovarian cancer (HGSOC) treatment-naïve and post-neoadjuvant chemotherapy (NACT) pairs identified 15 non-tumour clusters (Figs. 1A and S1B, C, clinical information in Table S1, GSE165897). To understand the changes in non-tumour cell states attributed to NACT, we modelled the differences on a K nearest neighbours (KNN) graph (k = 30) [16] (Fig. S1D). A total of 1437 differentially abundant neighbourhoods (896 upregulated and 541 downregulated in NACT) were identified at 10% FDR (Fig. 1A, B). Differential gene analysis of positively and negatively correlated neighbourhoods showed that T-cell receptor (TCR) signalling (*JUN, GNLY, CD160*, and *ID2*) was overactivated in the T subset, whereas the cell cycle pathway was impaired, indicating that proliferative ability was damaged following NACT (Figs. 1C, D and S1E). Differential gene analysis of macrophages showed that metabolism (*CD36, IDO1, SLC22A2*, and *SLC38A9*), transcriptional regulation (*KLF2 and JMJD1C*), cytokine secretion, and prostaglandins (*PTGES, PTGS1, TBXAS1*, and *AKR1C3*) were prominently induced by NACT (Figs. 1E, F and S1F). Moreover, the activation of cytokine and chemokine secretion upon NACT treatment was also observed in fibroblasts (Fig. 1G, H). These results indicate that NACT induces immune-activated microenvironment formation and partially affects T-cell proliferation.

### Dissecting cell type-specific and NACT-specific transcriptional heterogeneous pathways

Because strong intercellular heterogeneity obscures patterns of transcriptional variation across tumours, we performed a generalised binary covariance decomposition (GBCD) analysis. Interestingly, gene expression programme 7 (GEP7, including *CD24, S100A1, S100A13, S100A10, HSPB*) and GEP8 (*RPLs, RPSs*) were largely active in tumour cells from post-NACT patients (Fig. S2A–C), indicating that some subsets of tumour cells undergo significant translational remodelling after NACT. GBCD analysis of the CAF subset revealed that GEP19 (HLA-A/B/C/E and B2M) was induced following NACT (Fig. S2D, E), suggesting that NACT promotes the antigen-presenting ability of cancer-associated fibroblasts (CAFs). For immune cells, GBCD identified a macrophage-specific GEP (GEP6) that was not significantly altered upon NACT treatment (Fig. S2F, G). Moreover, we performed GBCD on non-tumour cells and found that GEP7, GEP8, and GEP15 were active in the post-NACT group (Fig. S2H). Antibody rearrangements (*IGHG4, IGLVs*), immune cell chemotaxis (*CCL3/4/18, CXCL1/2/3/8*), and pro-inflammatory factors (*IL1B, IL6, GZMB, IL11*) were extensively induced (Fig. S2I–K). These analyses show that NACT promotes a pro-inflammatory transition in the tumour microenvironment, which is not limited to immune cells.

### Tensor decomposition exploring the coordinated transcriptional variation among different interacting cell types

**Fig. 2:**
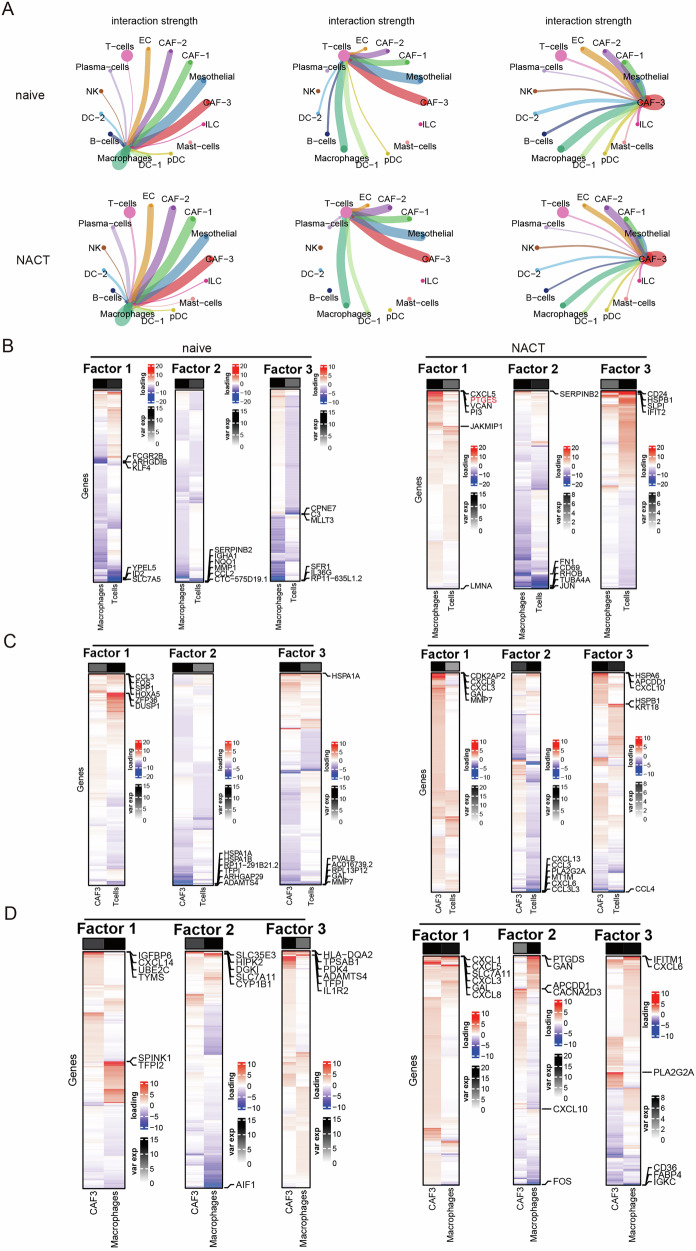
Remodelling of intercellular communication and transcriptional programmes in the tumour microenvironment following NACT. **A** Circos plot showing predicted intercellular communication networks based on interaction strength across macrophages (left), T cells (middle), CAF-3 cells (cancer-associated fibroblast-3, right), and other cell subsets in treatment-naïve (top) and post-NACT (bottom) tumours. Notable changes in signalling strength and partner rewiring were observed, particularly in interactions involving CAF-3 cells, macrophages, and endothelial cells. **B** Gene loading heatmaps for factors 1–3, displaying significant genes in naïve and NACT samples only. Coordinated interaction patterns between macrophages and T cells were analysed using single-cell Interpretable Tensor Decomposition (scITD). **C** Gene loading heatmaps for factors 1–3, displaying significant genes in naïve and NACT samples only. Coordinated interaction patterns between CAF3 and T cells were analysed using scITD. **D** Gene loading heatmaps for factors 1–3, displaying significant genes in naïve and NACT samples only. Coordinated interaction patterns between CAF3 and macrophages were analysed using scITD.

Considering that cellular interactions constitute the foundation of the tumour microenvironment, we inferred cell–cell communication using CellChat [17]. NACT obviously affected the interaction strength of DC and B cells, reduced the interaction strength between T cells and DC, but increased the interaction strength between macrophages and T cells, as well as between T cells and CAF-3 cells (Figs. 2A and S3). To further identify the patterns of coordinated cellular activity among these cell types following NACT, we performed scITD analysis based on Tucker tensor decomposition [18], and decomposed the dataset into three factors. Regarding the interaction between macrophages and T cells, NACT-induced *CXCL5, PTGES, CD24* and *IFIT2* expression while reducing *CD69* and *JUN* expression (Fig. 2B). Regarding the interaction between CAF-3 cells and T cells, NACT-induced *CXCL3/8/10* expression (Fig. 2C). These analyses confirmed that NACT facilitated T-cell attachment. However, scITD analysis of the interaction between macrophages and CAF-3 cells indicated that NACT-induced *CXCL1/5/6/8* and *PTGDS* expression (Fig. 2D). As mentioned before, prostaglandins, including PTGES and PTGDS, have been shown to impair the intratumoural orchestration of immune responses [19, 20]. We doubt whether NACT-induced prostaglandin increases mediate subsequent treatment resistance.

### Tumour cells directly induce phenotype switch of immune cells after cisplatin treatment

**Fig. 3:**
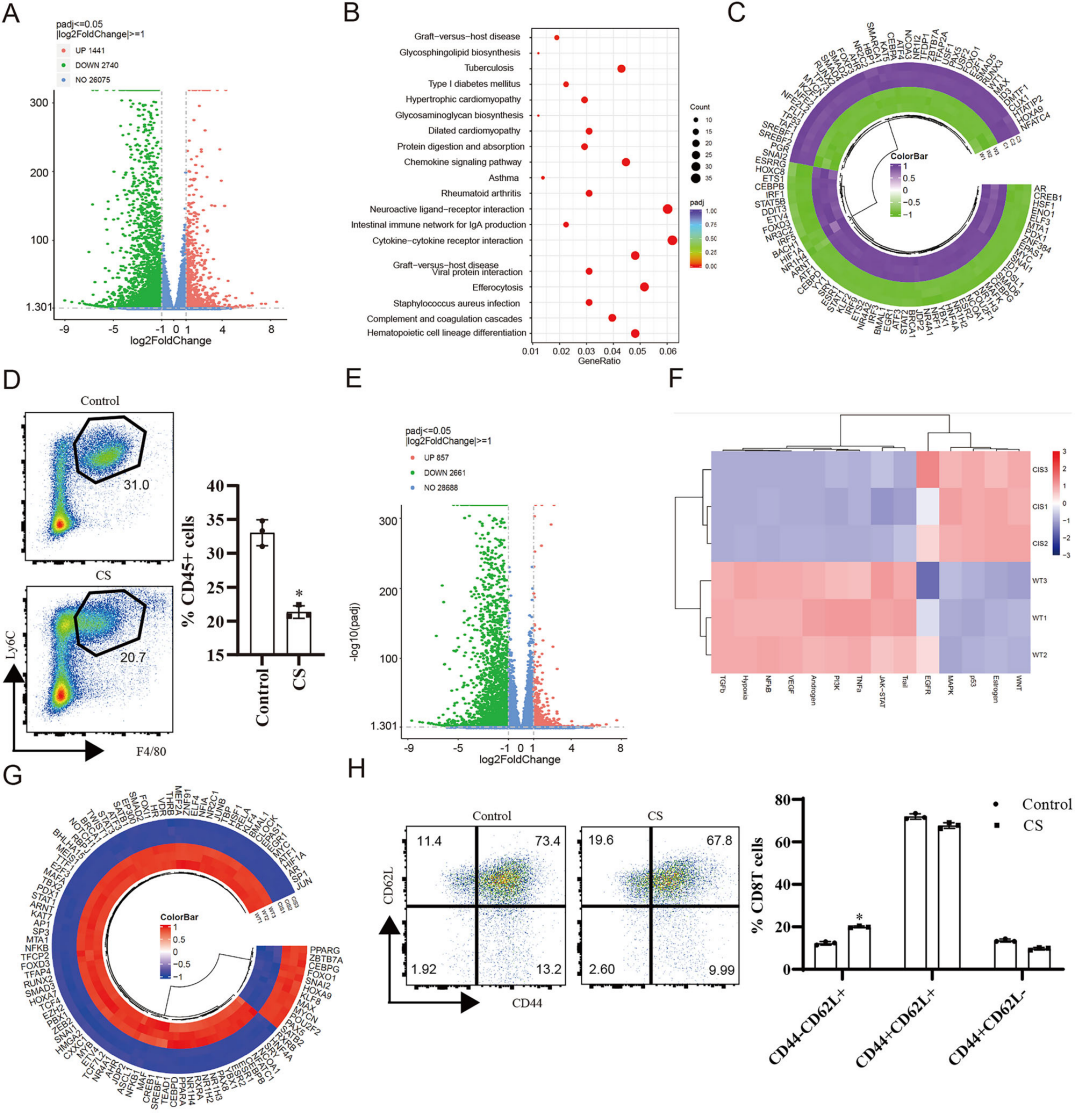
Cisplatin-induced tumour cells promote immunosuppressive characteristics of immune cells. **A** Volcano plot of differentially expressed genes in monocytes between cisplatin (CS)-treated and control groups, highlighting significantly upregulated (red) and downregulated (green) genes (adjusted *p* < 0.05, |log_2_FC| > 1). **B** Kyoto Encyclopedia of Genes and Genomes (KEGG) pathway enrichment of significantly dysregulated genes, with dot size representing gene count and colour representing the *p*-adjusted value. **C** Circo-heat map plot of transcription factor (TF) enrichment scores based on upregulated genes, highlighting shifts in TF activity following cisplatin treatment. **D** Flow cytometric quantification of F4/80⁺CD11b⁺ macrophages from control and CS-treated groups. Representative plots and statistical summary (*n* = 3, *p* < 0.05). **E** Volcano plot of differentially expressed genes in T cells between cisplatin (CS)-treated and control groups, highlighting significantly upregulated (red) and downregulated (green) genes (adjusted *p* < 0.05, |log_2_FC| > 1). **F** Heatmap of selected immune-related pathway enrichment analysis across independent samples between the CS-treated and control groups (*n* = 3 per group). **G** Circos heatmap plot of TF enrichment scores based on upregulated genes, highlighting shifts in TF activity following cisplatin treatment. **H** Flow cytometry analysis of CD8^+^ T-cell activation status (CD44^–^ CD62^+^ ratio) in the control and CS-treated groups. The right panel shows the qualified results (*n* = 3, *p* < 0.05).

We performed in vitro co-culture experiments with cisplatin-pretreated ID8 cells and bone marrow-derived macrophages (BMDMs) or T cells to evaluate the role of tumour cells in regulating the phenotypic transformation of immune cells. Transcriptome sequencing indicated 1441 upregulated and 2740 downregulated genes in BMDMs after inoculation (Fig. 3A). Immunity and inflammatory pathways, including chemokine signalling and cytokine–cytokine receptor interactions, were induced in BMDMs (Fig. 3B). However, interferon-response signalling (*STAT1, STAT2, IRF9*) and macrophage differentiation potential were reduced following inoculation (Fig. 3C, D). Upon inoculation with T cells, 2661 genes were downregulated, and 857 genes were upregulated (Fig. 3E). The JAK-STAT and NF-κB pathways were markedly inhibited in T cells following co-culture (Fig. 3F), which was also confirmed by TFs analysis and flow cytometry detection (Fig. 3G, H). Although cisplatin therapy delayed disease progression in an ID8 intraperitoneally-injected mouse model (Fig. S4A), but upregulated prostaglandin secretion (Fig. S4B, C). Moreover, the frequencies of MDSCs (PDL1^+^CD11b/Ly6C^+^) and exhausted CD8^+^T (PD1^+^TIM3^+^) increased following cisplatin treatment (Fig. S4D–F). These findings suggest that cisplatin treatment induces a pro-inflammatory state in the TME to a limited extent.

### NACT-specific tumour cell heterogeneity and co-regulatory network analysis

To understand the dynamic co-expression networks within ovarian cancer cells after NACT, we conducted high-dimensional weighted gene co-expression network analysis (hd-WGCNA) [21]. The co-expression networks identified by hd_WGCNA are based on transformed pairwise correlations of input gene features [22, 23]. After selecting the optimal soft-power threshold (*n* = 10), we constructed 16 modules (M1-M16, co-expression modules) among the cancer cells (EOC_C1-EOC_C12) (Fig. S5A–C). Module distribution analysis showed M6 was specific to EOC_C7, whereas M3 and M5 were present in multiple cancer cells subsets (Fig. S5D). In particular, M2, M4-M7, M13, and M16 were enriched in the post-NACT group compared to the naïve group (Fig. S5E). Gene ontology (GO) enrichment analysis indicated that the naïve M9 module was enriched in the negative regulation of interferon type II, whereas the NACT-related M13 module was enriched in the regulation of PRR and Myd88-dependent toll-like receptor (TLR) signalling (Fig. S5F, G). This suggests that NACT induces pro-inflammatory responses in cancer cells. Next, we mapped the dynamic network topological overlap matrix of the top hub genes from each module onto a two-dimensional manifold. We found that M4 highly enriched *PTGES, S100A9, MUC4* and *LAMA5* were closely related to M1 (enriched *RPL13A, PRL19, PRL28*) and M11 (enriched *CDK6, MAFB, CALR, ADRM1*) (Fig. S5H, I). These results clarify that M4-containing *PTGES* is strongly related to translational and transcriptional remodelling after NACT. Moreover, transcription factor spectrum analysis indicated TP53 activity was reduced, whereas STAT3, IRF5, and HSF2 activities were induced in EOC_C1. (Fig. S5J). GRHL2, ZNF140, and HSF2 activities were induced in EOC_C8 (Fig. S5K), and ZNF140 and HSF2 activities were both induced in EOC_C11 and EOC_C12 (Fig. S5L–N). Notably, ZNF140 and HSF2 activities were mostly upregulated in NACT-induced cancer cell subsets, indicating that these may be potential therapeutic targets for cancer recurrence.

### Cisplatin therapy induces an immunosuppressive microenvironment

**Fig. 4:**
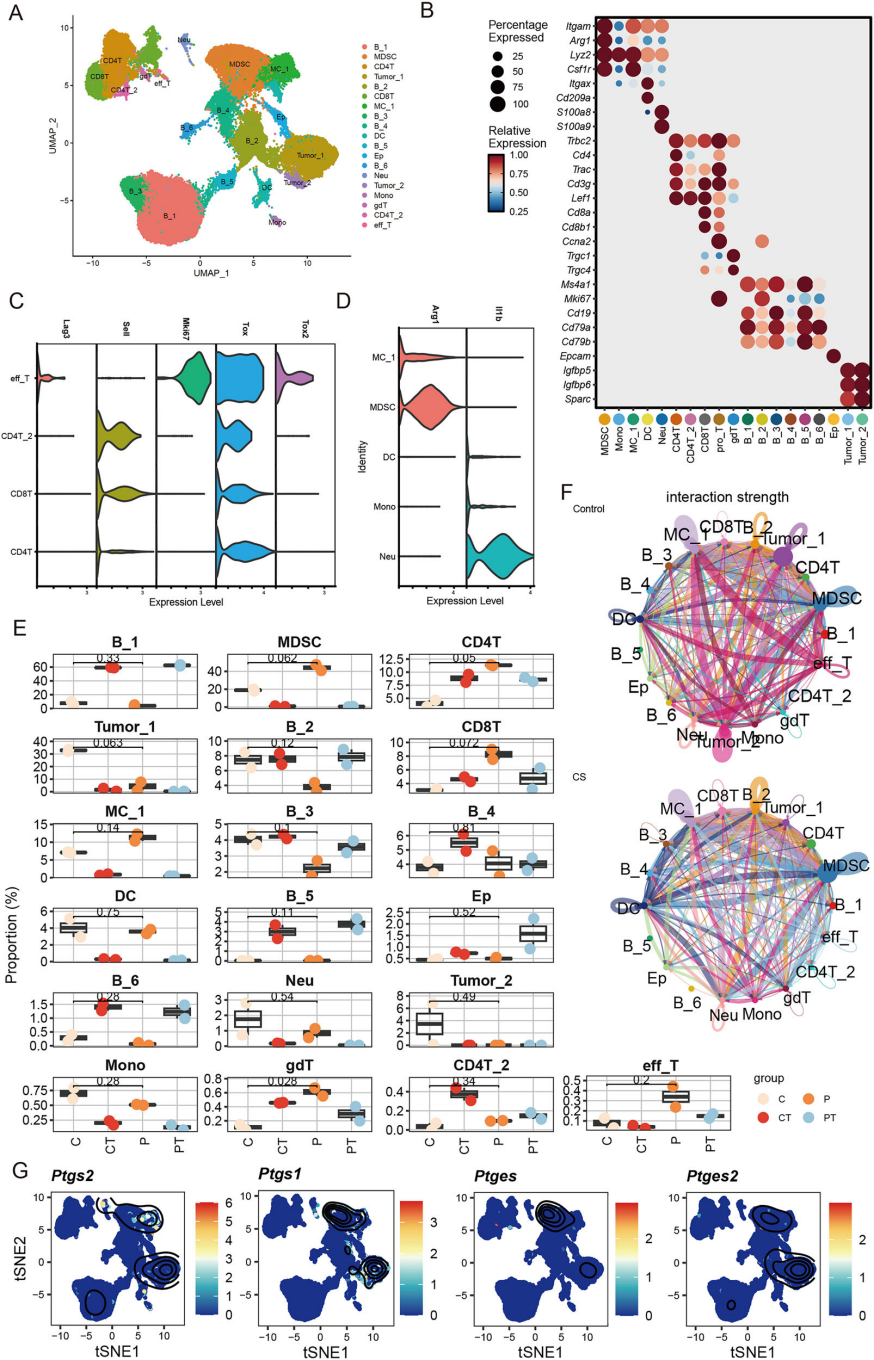
Single-cell transcriptomic profiling of immune cells reveals dynamic remodelling and suppressive signalling post-chemotherapy. **A** Uniform manifold approximation and projection (UMAP) embedding of single-cell transcriptomes from immune cells with clusters annotated by cell type (e.g. CD4^+^ T-cells, myeloid-derived suppressor cells [MDSCs], and B cells). **B** Dot plot showing the expression of key immune-related genes across the immune subtypes. Dot size indicates the percentage of cells expressing each gene; the colour scale shows the relative expression. Violin plots showing the expression of marker genes across T cells (**C**) and myeloid cell subsets (**D**). **E** Boxplots comparing the proportions of immune cell clusters across the four groups. **F** Cell–cell interaction networks inferred for the control and chemotherapy-treated groups. The edge thickness represents the strength of the interaction. Notable rewiring was observed in the MDSC–T-cell and tumour-B cell interactions. **G** tSNE expression density plots for *Ptgs2*, *Ptgs1*, *Ptges*, and *Ptges2*, indicating that prostaglandin signalling was mainly enriched in myeloid and tumour cells.

We speculate whether prostaglandins were linked to immunosuppressive features upon NACT. To test this hypothesis, we performed scRNA-seq using the ID8 intraperitoneal injection model. After rigorous quality control, we obtained 60,744 high-quality cells from four groups (C/P/CT/PT and control/cisplatin/control metastasis/cisplatin metastasis), including peritoneal irrigation fluid and metastasis with or without cisplatin therapy. Uniform manifold approximation and projection (UMAP) plots further partitioned the 19 clusters, including B cells (*Ms4a1*, and *Cd79a, Cd19*), T cells (*Trbc2*, *Trac*, and *Cd3g*), myeloid cells (*Itgam, Lyz2, Csf1r, Itgax, S100a8*, and *S100a9*), tumour cells (*Igfbp5*, *Igfbp6*, and *Sparc*), and epithelial cells (*Epcam*) (Figs. 4A, B and S6A, B). As expected, cisplatin decreased the proportion of tumour cells but increased the proportion of total T cells, effector T cells and MDSCs (Figs. 4C–E and S6C, D). Surprisingly, B cells were the most abundant immune cells in the metastatic group, comprising > 50% of the immune compartment (Fig. S6D). The strength of communication among MC_1 (macrophages), DC (dendritic cells), and eff_T (effector T cells), or among DC, MC, and tumour cells (Tumour _1, Tumour _2) was markedly reduced following cisplatin therapy (Fig. 4F). Conversely, the strength of communication among MDSC, DC, and eff_T cells was induced after cisplatin therapy (Fig. 4F). Similarly, pseudo-time analysis demonstrated that cisplatin completely promoted MDSC differentiation, whereas macrophage and MDSC differentiation occurred only in the control group (Fig. S6E, F). Next, we examined the expression of prostaglandin family members and found that Ptgs1/2 and Ptges1/2 were highly expressed in both MDSC and tumour cells, which is consistent with the above findings (Fig. 4G).

### Cisplatin promotes *PTGES* transcription dependent on NF-*κ*B pathway

**Fig. 5:**
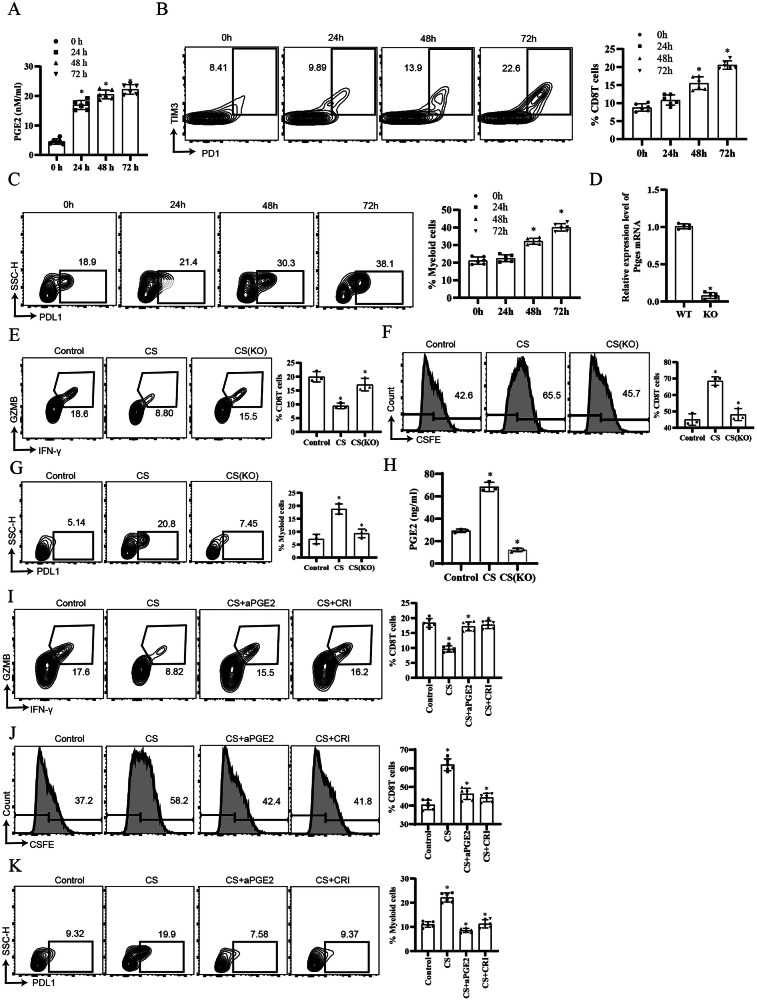
Cisplatin-induced PGE2 (prostaglandin E2) secretion promotes anti-tumour immune dysfunction. **A**–**C** Female mice were intraperitoneally injected with ID8 (stably expressing the luciferase gene promoter) cells for 2 weeks, followed by cisplatin treatment. ELISA (enzyme-linked immunosorbent assay) analysis of prostaglandin E2 levels in peritoneal irrigation fluid (**A**) at the indicated time points. Flow cytometric analysis of PD1^+^TIM3^+^CD8^+^T cells (**B**) and PD-L1^+^ myeloid cells (**C**) in tumours (*n* = 6, *p* < 0.05). **D**–**H** Wild-type (WT) or *Ptges* knockout (KO) ID8 cells were co-cultured with activated CD8^+^T cells or monocytes. *Ptges* mRNA in WT and KO ID8 cells was detected using QRT-PCR (Quantitative Real-time polymerase chain reaction) (**D**). Flow cytometric analysis of GZMB^+^IFN-γ^+^CD8^+^T (**E**) CSFE^+^CD8^+^T cells (**F**) and PD-L1^+^ myeloid cells (**G**). The prostaglandin E2 levels in the supernatant were detected by ELISA (**H**) (*n* = 3, *p* < 0.05). **I**–**K** ID8 cells treated with anti-PGE2 antibody (αPGE2) or CRI (crisdesalazine) were co-cultured with activated CD8^+^T cells or monocytes. Flow cytometric analysis of GZMB^+^IFN-γ^+^CD8^+^T (**I**), CSFE^+^CD8^+^T cells (**J**) and PD-L1^+^ myeloid cells (**K**) (*n* = 6, *p* < 0.05).

**Fig. 6:**
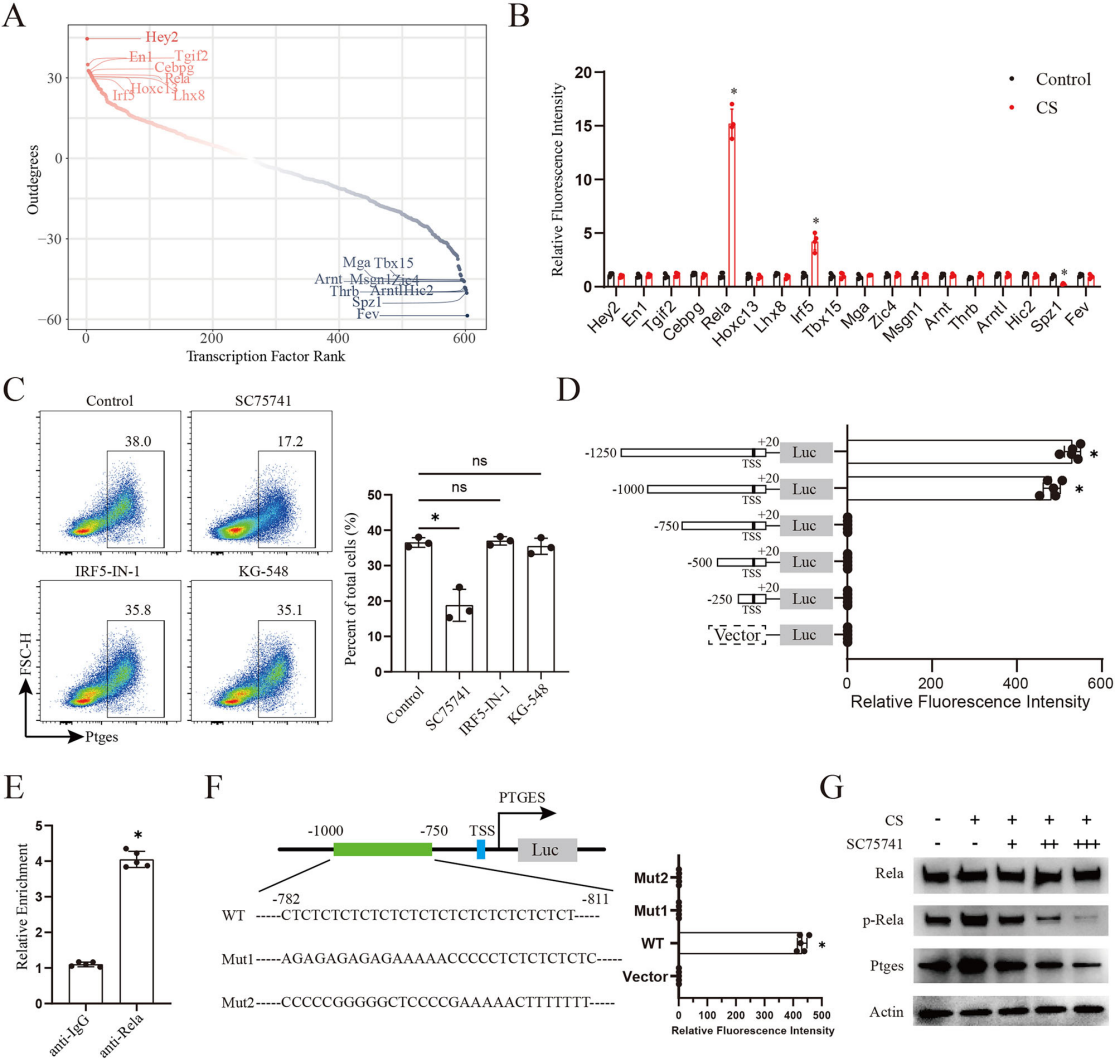
Cisplatin promotes PTGES transcription through RELA-dependent transcriptional activation. **A** SCORPION (Single-Cell Oriented Reconstruction of PANDA Individually Optimized Gene Regulatory Networks) analysis of single-cell RNA-seq data from ovarian cancer samples before and after cisplatin treatment to infer changes in transcription factor activity in tumour cells. Transcription factors are ranked according to differential activity, with the most significantly altered factors highlighted. **B** Transcription factor activation profiling of candidate transcription factors binding to the Ptges promoter in ID8 cells following 24 h cisplatin treatment (10 μM) (*n* = 3, *p* < 0.05). **C** ID8 cells were treated with inhibitors targeting Rela, Irf5, or Arnt, and PTGES expression was analysed by flow cytometry (*n* = 3, *p* < 0.05). **D** Dual-luciferase reporter assays using serially truncated *Ptges* promoter constructs were performed to determine Rela-mediated transcriptional activation (*n* = 5, *p* < 0.05). **E** ChIP–qPCR analysis confirming RELA enrichment at the −1000 to −750 bp region of the *Ptges* promoter identified in (**D**) (*n* = 5, *p* < 0.05). **F** Site-directed mutagenesis of predicted RELA-binding motifs within the *Ptges* promoter (based on Animal TFBDs predictions), followed by dual-luciferase reporter assays to evaluate the requirement of these motifs for RELA-dependent transcriptional activation (*n* = 5, *p* < 0.05). **G** ID8 cells were treated with cisplatin (10 μM) and/or the RELA inhibitor SC75741 (5 μM, 10 μM, 20 μM) for 24 h, and the protein levels of Rela, phosphorylated Rela (p-Rela) and PTGES were detected by immunoblotting.

To elucidate the causal relationship among cisplatin treatment, PGE2 production, and anti-tumour immune dysfunction, we firstly quantified PGE2 secretion in an ID8 intraperitoneally-injected mouse model. Notably, increased PGE2 production preceded the upregulation of exhausted CD8^+^T and MDSCs (Fig. 5A–C). Genetic ablation of *Ptges*, neutralisation of PGE2 using an anti-PGE2 antibody, or pharmacological inhibition of prostaglandin E synthase (PGES) with crisdesalazine (CRI) partially rescued cisplatin-induced impairments in CTL cytotoxicity and proliferation, as well as MDSC differentiation (Fig. 5D–K). To further explore the molecular mechanism, we analysed transcription factor activity in tumour cells using scRNA-seq and identified significant upregulation of Hey2, En1, and Rela (Fig. 6A). Transcription factor activation profiling further revealed a marked increase in the promoter-binding activity of Rela (Fig. 6B). Flow cytometric analysis revealed that inhibition of Rela—but not Irf5 or Arnt—significantly attenuated cisplatin-induced *Ptges* expression (Fig. 6C). Dual-luciferase reporter assays identified critical RELA-responsive elements within the −1000 to −750 bp region of the Ptges promoter (Fig. 6D). Consistently, chromatin immunoprecipitation followed by quantitative PCR confirmed significant enrichment of Rela at this promoter region (Fig. 6E). To validate the functional relevance of these elements, site-directed mutagenesis targeting the predicted Rela-binding motifs (based on Animal TFBDs predictions) was performed, which completely abolished Rela-mediated transcriptional activation of the *Ptges* promoter (Fig. 6F). Finally, immunoblot analysis showed that cisplatin treatment markedly enhanced Rela phosphorylation and Ptges protein expression, both of which were effectively suppressed by the Rela inhibitor SC75741 (Fig. 6G).

### Inhibition of prostaglandin E synthase boosts antitumour immunity under cisplatin treatment

**Fig. 7:**
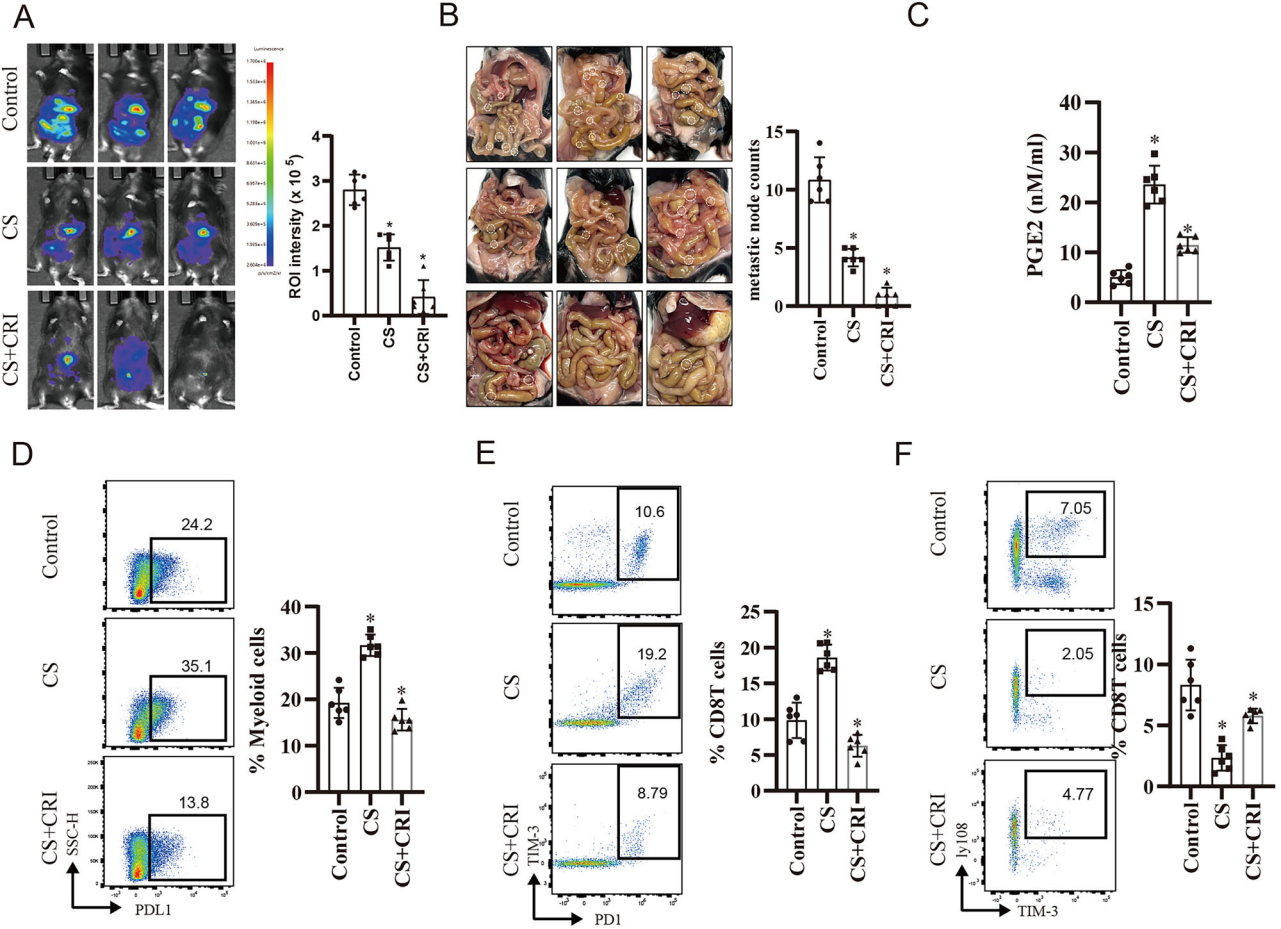
Combined chemotherapy and PGES (prostaglandin E synthase) inhibition reduces tumour burden and reverses immunosuppression. **A**, **B** Female mice were intraperitoneally-injected with luc-ID8 (stably expressing the luciferase gene promoter) cells for 2 weeks. Subsequently, cisplatin or cisplatin plus CRI (crisdesalazine) was administered twice per week. In vivo imaging was performed on 4th week. The right panel shows the results. The representative pictures are shown (**A**) (*p* < 0.05). Representative images of metastatic nodules in the peritoneal cavity from each treatment group, with bar graph quantifying the number of visible metastases. Combination treatment further reduced the metastatic spread (*n* = 6, *p* < 0.05). **C** PGE2 concentrations in tumour supernatants, as determined by ELISA. CS treatment increased PGE2 production, which was significantly reduced by CRI treatment (*n* = 6, *p* < 0.05). **D** Flow cytometric analysis of PD-L1^+^ myeloid cells in tumours. CS treatment increased the proportion of immunosuppressive PD-L1^+^ myeloid cells, which was normalised by CS + CRI therapy (*n* = 6, *p* < 0.05). **E** Exhausted CD8+ T cells were significantly increased in the CS group but were partially restored following CRI treatment (*n* = 6, *p* < 0.05). **F** The stemness of CD8+ T cells was significantly decreased in the CS group but was partially restored following CRI treatment (*n* = 6, *p* < 0.05).

To investigate the role of prostaglandins in cisplatin-induced immunosuppressive microenvironment formation, we used CRI or αPGE2 in an ID8 intraperitoneal injection mouse model. Notably, the combined application of cisplatin and CRI or αPGE2 delayed tumour progression, metastasis, and prostaglandin secretion compared with cisplatin monotherapy (Figs. 7A–C and S7C, D). Immune cell infiltration analysis based on peritoneal irrigation fluid indicated that CRI or αPGE2 reversed cisplatin-induced MDSC (PDL1^+^CD11b^+^ or PDL1^+^Ly6C^+^) infiltration and rescued cisplatin-induced CD8^+^T exhaustion, differentiation (PD1^+^TIM3^+^CD8T), and stemness (ly108^+^TIM3^+^CD8T) impairment (Figs. 7D, E and S7E–G, gating strategies in Fig. S7A, B). However, a combination therapy plan including cisplatin and paclitaxel (C+T) has always been chosen for NACT. We also tested another PGES inhibitor (AGU654) using this therapeutic mouse model. Consistent with cisplatin monotherapy, AGU654 facilitated C+T-induced inhibition of tumour progression (Fig. S7H, I), decreased prostaglandin secretion, and reduced MDSCs and exhausted CD8^+^T infiltration (Fig. S7J–L). These results suggest that prostaglandins mediate the formation of an immunosuppressive microenvironment during NACT.

## Discussion

Advanced ovarian cancer remains a major therapeutic challenge owing to high relapse rates and the development of platinum resistance. This study aimed to clarify how NACT remodels the TIME and whether prostaglandin inhibition mitigates NACT-induced immunosuppression. Our data show that NACT induces a transient pro-inflammatory shift in the tumour microenvironment, followed by prostaglandin-mediated immunosuppression. Specifically, we observed (i) transcriptional remodelling of the tumour and stromal compartments, (ii) increased MDSC infiltration and CD8^+^ T-cell exhaustion post-NACT, and (iii) reversal of these effects upon PGES inhibition in preclinical models. To our knowledge, this is the first study to demonstrate, using integrated single-cell transcriptomics and functional assays, that NACT-induced prostaglandin production by both tumour cells and MDSCs contributes directly to CD8^+^ T-cell exhaustion and an immunosuppressive microenvironment in ovarian cancer.

NACT has become a cornerstone in the management of advanced ovarian cancer, particularly in patients deemed unsuitable for primary cytoreductive surgery. Beyond its role in reducing the tumour burden and facilitating surgical resection, emerging evidence highlights its profound impact on reshaping the tumour immune microenvironment (TIME), which may influence therapeutic outcomes and resistance mechanisms. For example, NACT may transiently enhance immunogenicity by exposing tumour antigens, but concurrently upregulate immune checkpoints (e.g. PD-1/PD-L1) [24]. Importantly, our study demonstrates that cisplatin-induced prostaglandin upregulation contributes to the infiltration of PD-L1⁺ MDSCs. We therefore propose that combining cisplatin with prostaglandin inhibition may enhance the efficacy of immune checkpoint blockade, a hypothesis that warrants further investigation. Furthermore, NACT remodels the ovarian cancer TIME through complex interactions involving T-cell exhaustion, macrophage polarisation, and metabolic adaptation. Although these changes may initially enhance the treatment efficacy, residual immunosuppressive networks often drive relapse [25]. The heterogeneity of the TIME post-NACT necessitates personalised approaches. For instance, hyperthermic intraperitoneal chemotherapy (HIPEC) during cytoreduction surgery may augment local immune activation in selected patients. Moreover, integrating single-cell transcriptomic profiling (e.g. to identify IFN-responsive T cells or RTM-like macrophages) could help stratify patients for immunotherapy or PARP inhibitor maintenance. Clinical trials exploring NACT combined with immune checkpoint inhibitors or eTreg-targeted therapies (e.g. CCR8 blockade) are warranted to exploit these mechanistic insights [26, 27].

Single-cell analyses revealed that ovarian cancer ascites, a common feature of advanced disease, serve as a reservoir for memory T cells (Tcm/Tem) that infiltrate primary and metastatic tumours. These cells exhibit TCR clonotypes shared with terminally exhausted T cells (Tex) in tumour tissues, suggesting a dynamic replenishment mechanism. After NACT, ascites-derived CD8^+^GZMK^+^ Tem cells may migrate to the tumour sites and transition into Tex cells, thereby perpetuating immune dysfunction [28]. Consistent with this phenomenon, the frequency of exhausted CD8^+^T cells increased prominently following cisplatin treatment in the mouse model. In contrast, TAMs and MDSCs were critically remodelled by NACT. M2-polarised macrophages promote cisplatin resistance [29]. By regulating adenosine synthesis, CD39^+^MDSCs facilitate cisplatin resistance in ovarian cancer cells [30]. Flow cytometry analysis also revealed that cisplatin administration promoted MDSCs formation, which is necessary for CD8^+^ T-cell exhaustion differentiation. Subsequently, in vivo and in vitro experiments suggested that prostaglandin inhibition boosts cytotoxic T lymphocyte (CTL) cytotoxicity and partially rescues the suppressive microenvironment in cisplatin-stimulated ovarian cancers. However, prostaglandin-mediated immune suppression may also contribute to patient relapse.

This study had some limitations. First, the interaction networks between tumour cells and multiple immune cells under NACT should be further demonstrated by in vitro experiments using patient-derived organoids. Second, the frequency of CD4T cells also increased in the cisplatin group. As important helpers, CD4T cells bridge the antigen presentation system and CTLs; this unexpected phenomenon warrants further investigation. Third, the ID8 syngeneic model employed in this study does not fully recapitulate the biological complexity of human high-grade serous ovarian cancer (HGSOC). Patient-derived xenografts (PDXs) or other genetically engineered models will be required to validate and extend our findings. This study integrated scRNA-seq analysis with mouse models to identify prostaglandin-mediated immunosuppression following NACT. Targeting prostaglandins alleviates MDSC-mediated inhibition of antitumour immunity, thereby strengthening the efficacy of cisplatin or cisplatin/paclitaxel therapy, which may provide a novel therapeutic strategy for treating ovarian cancer recurrence.

## Methods

### Cell culture

Mouse spleen-derived primary T cells were isolated from C57BL/6 mice (6–8 weeks old) using the Pan T-Cell Isolation Kit II (#130-095-130, Miltenyi Biotec., Germany), and seeded in complete medium including ALyS505N-0 medium (#1020P10, Baso, China), 10 ng/mL IL-2 (#212-12-20UG, ThermoFisher, USA), and 10% FBS (#A5669701, Gibco, USA) in a humidified 5% CO_2_ atmosphere at 37 °C. Before co-culture with ID8 cells, T cells were pre-activated with anti-CD3/28 magnetic beads (1:2000 dilution, #11456D, ThermoFisher, USA) for 72 h. The mouse ovarian cancer cell line ID8 was cultured in DMEM (#11965118, Gibco, USA) supplemented with 10% FBS at 37 °C under 5% CO_2_. Mouse bone marrow-derived monocytes were isolated from C57BL/6 mice (6–8 weeks old) and seeded in complete medium, including RPMI 1640 medium (#12633020, Gibco, USA) and 10% FBS, in a humidified 5% CO_2_ atmosphere at 37 °C.

### RNA-sequence and data analysis

Following co-culture with ID8 cells, monocytes and T cells were collected. Total RNA was extracted using the TRIzol reagent (#15596018CN, Invitrogen, USA). RNA-seq was performed using the HiSeq 6000 system (Illumina, USA) at Novogene (Beijing, China). Raw sequencing data (FASTQ format) were subjected to quality control analysis. Low-quality reads, adaptor-containing reads, and reads containing more than 10% unknown nucleotides were removed. Clean reads were used for all downstream analyses after calculating the quality metrics. We employed LifeScope v2.5.1 to align the reads to the genome, generate raw counts corresponding to each known gene, and calculate both raw counts and reads per kilobase per million. Differential expression analysis (between two experimental conditions, including biological replicates) was performed using DESeq2 (v1.20.0) based on an adjusted *p* value < 0.05 and fold change ≥2 (significant gene standard).

### MiloR analysis

Based on a cell–cell similarity structure, Milo leverages the flexibility of generalised linear models, thereby accounting for disturbances, including complex experimental designs (such as the inclusion of nuisance and technical covariates) and batch effects across different samples. Moreover, by modelling cell states as overlapping neighbourhoods, miloR analysis aims to accurately pinpoint perturbed cellular states, enabling the exploration of underlying molecular programmes. MiloR analysis was performed as described previously (https://github.com/MarioniLab/miloR). Briefly, the count matrix extracted from scRNA-seq was refined and sampled to capture the continuous trajectories. Differential abundance testing was performed by assigning cells to partially overlapping neighbourhoods using the k-nearest neighbour graph method. This analysis identifies perturbations obscured by the distribution of cells into clusters. The main parameters used were as follows: (1) buildGraph (k = 30, d = 20), (2) makeNhoods (prop = 0.2, k = 30, d = 20, refined = TRUE), and calcNhoodDistance (d = 20).

### Generalised binary covariance decomposition analysis

Strong intratumor heterogeneity obscures the subtle patterns shared across tumours. Generalised binary covariance decomposition (GBCD) is used to decompose transcriptional heterogeneity into interpretable components, including cell-type- and sample-specific components relevant to disease subtypes. GBCD analysis was performed as previously described (https://github.com/stephenslab/gbcd). In this study, we used GBCD to refine the characterisation of gene expression programmes relevant to NACT. The main parameters used were as follows: (1) Kmax = 12; (2) maxtier1 = 100; (3) maxtier2 = 50; (4) maxiter3 = 50; (5) prior_family = ‘generalised _binary’; and (6) scale = 0.04.

### Cellchat analysis

Based on a manually curated database of literature-supported ligand-receptor interactions, CellChat was used to infer cell–cell communication and ligand-receptor interactions across different treatments or samples. CellChat analysis was performed as previously described (https://github.com/sqjin/CellChat). Briefly, the inferred cell–cell communication networks among non-parenchymal cells in the naïve and NACT groups were quantitatively characterised and compared by combining pattern recognition, manifold learning, and social network analysis approaches. The main parameters were as follows: (1) subsetDB (search = ‘Secreted Signalling’) and (2) filterCommunication (min.cells = 10).

### Single-cell Interpretable Tensor Decomposition analysis

Single-cell Interpretable Tensor Decomposition (scITD) aims to identify common axes of inter-individual variation by considering joint expression variation across multiple cell types, based on the premise that higher-level biological processes involve the interactions of multiple cell types across heterogeneous samples. scITD analysis was performed as previously described (https://github.com/kharchenkolab/scITD). In this study, we aimed to detect the joint patterns of coordinated action among macrophages, T cells, and CAF-3 cells. The main parameters used were as follows: (1) param_list<-initialise _params (ncores = 30, rand_seed = 10); (2) form_tensor (donor_min_cells = 5, norm_method = ‘trim’, scale_factor = 5000, vargenes_method = ‘norm_var_pvals’, vargenes_thresh = 0.1, scale_var = TRUE, var_scale_power = 2); get_all_lds_factor_plots (use_sig_only = TRUE, nonsig_to_zero = TRUE, sig_thresh = 0.02, display_genes = FALSE, gene_callouts = TRUE,callout_n_gene_per_ctype = 3, show_var_explained = TRUE).

### Hd-WGCNA analysis

High-dimensional weighted gene co-expression network analysis (hd-WGCNA) was performed to identify context-specific co-expression networks across different cell types or treatments by identifying modules of highly co-expressed genes. High-dimensional weighted gene co-expression network analysis (hd-WGCNA) was performed as described previously (https://github.com/smorabit/hdWGCNA). Ovarian cancer cell subsets were extracted from scRNA-seq data and used to construct co-expression networks across cellular and spatial hierarchies. Modules of highly co-expressed genes were identified by statistical testing and subsequent transcription factor activity analysis. The main parameters were as follows: (1) SetupForWGCNA (gene_select = ‘fraction’, fraction = 0.05); (2) MetacellsByGroups (k = 25, max_shared = 20); (3) ConstructNetwork (power = 10); (4) TFBSTools::getMatrixSet (x = JASPAR2020, opts = list (collection = ‘CORE’, tax_group = ‘vertebrates’, all_versions = FALSE)). (5) AssignTFRegulons (strategy = ‘A’, reg_thresh = 0.01, n_tfs = 10).

### Flow cytometry

Peritoneal irrigation fluid was isolated from the mice and prepared as a single-cell suspension for analysis. Cultured monocytes or T cells were collected and washed with phosphate-buffered saline (PBS) before preparing single-cell suspensions. After centrifugation, the cells were incubated with Fc receptor blocking solution (cat# 553141; BD, USA), collected, and stained with fluorochrome-conjugated antibodies for 30 min at 4 °C in the dark. Finally, the cells were washed and analysed using a BD FACSCanto II flow cytometer (BD Biosciences). Data were analysed using FlowJo X software (TreeStar, USA). The antibodies used were as follows: FVS780 (cat#565388, BD, USA), V450 anti-CD45 (cat#75-0451-U100, clone 30-F11, Tonbo, USA), PE anti-F4/80 (cat#111603, clone W20065B, BioLegend, USA), BV510 anti-CD3e (cat#100233, clone 17A2, BioLegend, USA), Percp5.5 anti-CD8a (cat#65-0081-U100, clone 53-6.7, Tonbo, USA), FITC anti-PDL1 (clone 10F.9G2, Elabscience), PE-cy7 anti-PD1 (cat#109110, clone RMP1-30, BioLegend, USA), PE-cy7 anti-Ly6C (cat#128017, clone HK1.4, BioLegend, USA), FITC anti-CD44 (cat#156007, clone NIM-R8, BioLegend, USA), APC anti-CD62L (cat#161217, clone W18021D, BioLegend, USA), PE anti-TIM3 (#50-5870-U100, Tonbo), APC anti-ly108 (cat#134609, BioLegend, USA). All experiments were performed in triplicate.

### SCORPION analysis

SCORPION analysis was performed to infer transcription factor (TF) activity and regulatory network remodelling from scRNA-seq data. Raw count matrices were extracted from the RNA assay of Seurat objects and filtered to retain genes expressed in more than 10% of cells. Analyses were restricted to tumour cells, which were grouped according to treatment conditions. TF regulatory networks were inferred using the scorpion() function with mouse TF–motif annotations (mmTF) and a curated Mouse protein–protein interaction network (mmPPI). TF activity was quantified based on TF-target regulatory edge weights rather than TF expression levels, yielding a TF-by-gene regulatory matrix (regNet) for each sample. To ensure comparability across datasets, regulatory networks were harmonised by unifying TF and target gene sets across all samples, with missing TF-target pairs padded with zeros. Differential TF activity and TF-target edge weights between Responder and Nonresponder groups were evaluated using a generalised linear model-based framework, followed by F-tests and Benjamini-Hochberg correction for multiple hypothesis testing. TFs were ranked by the absolute sum of their regulatory weights (outdegrees), and statistical significance was defined at a false discovery rate (FDR) < 0.05. SCORPION analysis was conducted according to the original workflow as described in the published study (https://github.com/kuijjerlab/SCORPION), using default parameters.

### Transcription factor (TF) activation profiling

ID8 cells were collected following cisplatin treatment, and nuclear proteins were extracted. TF activation profiling was performed according to the manufacturer’s instructions (#FA-1016, Signosis, USA). Briefly, TF probes were incubated with nuclear extracts and/or promoter sequences, followed by separation of free and bound probes. The bound probes were subsequently eluted and hybridised, and fluorescence signals were detected using a chemiluminescence analyser.

### Dual-luciferase reporter assay

Dual-luciferase reporter assays were performed to assess Rela-mediated transcriptional regulation of the *Ptges* promoter. Briefly, genomic fragments of the mouse Ptges promoter with varying lengths were amplified by PCR and cloned into the pGL3-basic luciferase reporter vector. For promoter truncation analyses, a series of constructs encompassing defined regions upstream of the transcription start site were generated as indicated. Site-directed mutagenesis of the predicted Rela-binding motifs within the *Ptges* promoter was carried out using a PCR-based mutagenesis strategy, and all constructs were sequence-verified. ID8 cells were seeded into 96-well plates and transfected at approximately 70–80% confluency using Lipofectamine 3000 (Thermo Fisher Scientific, #L3000150, USA) according to the manufacturer’s instructions. Each well was co-transfected with 500 ng of *Ptges* promoter–driven firefly luciferase reporter plasmid and 20 ng of Renilla luciferase plasmid as an internal control. Where indicated, Rela expression plasmids or the corresponding empty vectors were co-transfected. Forty-eight hours after transfection, cells were lysed, and luciferase activities were measured using the Dual-Luciferase Reporter Assay System (Promega, #E1910, USA) on a luminometer. Firefly luciferase activity was normalised to Renilla luciferase activity to control for transfection efficiency, and relative luciferase activity was calculated by normalisation to the corresponding control group.

### Chromatin Immunoprecipitation-quantitative Polymerase Chain Reaction (ChIP-qPCR)

Chromatin immunoprecipitation followed by quantitative PCR (ChIP–qPCR) was performed to assess Rela binding to the *Ptges* promoter. Briefly, ID8 cells were treated as indicated and cross-linked with 1% formaldehyde at room temperature for 10 min. Cross-linking was quenched by the addition of 125 mM glycine for 5 min. Cells were subsequently washed with cold PBS and harvested for chromatin preparation. Cell pellets were lysed, and chromatin was sonicated to generate DNA fragments with an average size of approximately 200–500 bp. After clarification by centrifugation, equal amounts of chromatin were incubated overnight at 4 °C with an anti-RELA antibody or normal IgG as a negative control, followed by incubation with protein A/G magnetic beads. Immunoprecipitated chromatin was sequentially washed, eluted from the beads, and reverse cross-linked at 65 °C overnight. DNA was purified using a PCR purification kit (Qiagen, #1020P10, Germany) and subjected to quantitative PCR analysis using SYBR Green Master Mix. Primers were designed to amplify the −1000 to −750 bp region of the *Ptges* promoter. ChIP–qPCR signals were calculated as the percentage of input DNA and are presented as fold enrichment relative to the IgG control. The primer sequences were as follows: forward, 5′-ATGGTGTGTGTGTGTGTGTGT-3′; reverse, 5′-AAGAACAGTAATCAGGCCAAA-3′.

### scRNA-seq analysis

Female mice (6–8 weeks old) were intraperitoneally-injected with ID8 cells. Two weeks later, cisplatin was administered twice weekly to the chemotherapy (CS) group. For another 4 weeks, peritoneal irrigation fluid and metastatic nodes in the mesentery, omentum, and peritoneum were collected as primary and metastatic tumour samples, respectively. After washing twice with PBS, single-cell suspensions (1 × 10^5^ cells/mL) were prepared, and sequencing was performed using an Illumina NovaSeq 6000 (at least 100,000 reads per cell). The raw data and generated gene count data were analysed using the Cell Ranger v3.0.2 pipeline. Low-quality cells (empty droplets, multiplets, and cells with <200 or >6000 expressed genes) were excluded. The R package Seurat (v4.0.4, http://satijalab.org/seurat/) was used for quality control, and all subsequent analyses were based on a gene-barcode matrix containing barcoded cells and gene expression counts.

### Animal experiments

Female C57BL/6 mice (6–8 weeks old) were purchased from the Model Animal Research Center of Nanjing University (Nanjing, China). The mice were intraperitoneally-injected with Luc-ID8 cells. Two weeks later, cisplatin (2.5 mg/kg) or cisplatin (2.5 mg/kg)/Taxol (10 mg/kg) were injected intraperitoneally twice a week. Crisdesalazine or AGU654 was intraperitoneally injected at a dose of 3.3 mg/kg or 10 mg/kg twice per week starting at 2 days post cisplatin treatment. Inhalation anaesthetics were administered with 5% isoflurane in 100% oxygen at a constant flow rate of 1.0 L/min (lasting for 5 min) in an induction chamber. Mice were euthanized by carbon dioxide (CO₂) asphyxiation followed by cervical dislocation to ensure death. Peritoneal irrigation fluid was isolated for another 2 weeks and used for subsequent flow cytometric analyses. Randomisation was applied when the mice were intraperitoneally-injected with Luc-ID8 cells or other reagents. When mice were sacrificed for subsequent flow cytometric analysis, randomisation was also applied in assuring the mouse order. No blinding experiments were involved in this study due to the authors’ perceived low likelihood of bias in final readouts. A sample size of six mice per group was used in the animal experiments.

### Statistical analysis

All the experiments about the molecular and cellular studies were repeated in triplicate. For the animal experiments, a sample size of six mice per group was used. Data were presented as mean ± s.d., and were analysed using GraphPad Prism software (version 8.0). An unpaired two-tailed Student’s t-test was used to compare the two groups. One-way ANOVA without adjustments was used to compare multiple groups. *P* < 0.05 was considered statistically significant.

## Supplementary information

Table_S1

Figure S1

Figure S2

Figure S3

Figure S4

Figure S5

Figure S6

Figure S7

Supplementary Data

Uncropped western blots

## Data Availability

Raw RNA-seq and scRNA-seq data were deposited in the NCBI under accession numbers GSE302946/GSE302947 and GSE303696, respectively.

## Code availability

MiloR analysis was performed as described previously (https://github.com/MarioniLab/miloR). Generalised binary covariance decomposition analysis was performed as previously described (https://github.com/stephenslab/gbcd). CellChat analysis was performed as previously described (https://github.com/sqjin/CellChat). scITD analysis was performed as previously described (https://github.com/kharchenkolab/scITD). High-dimensional weighted gene co-expression network analysis (hd-WGCNA) was performed as described previously (https://github.com/smorabit/hdWGCNA). SCORPION analysis was conducted according to the original workflow as described in the published study (https://github.com/kuijjerlab/SCORPION). The R package Seurat (v4.0.4, http://satijalab.org/seurat/) was used for analysing scRNA-seq.
