# Supplementary figures and images for: Dynamic changes of the immune microenvironment in ovarian cancer following neoadjuvant chemotherapy
Source: Cell Death Discov. 2026 Mar 23;12:130. doi: 10.1038/s41420-026-03070-6 (PMC13039919; doi:10.1038/s41420-026-03070-6)

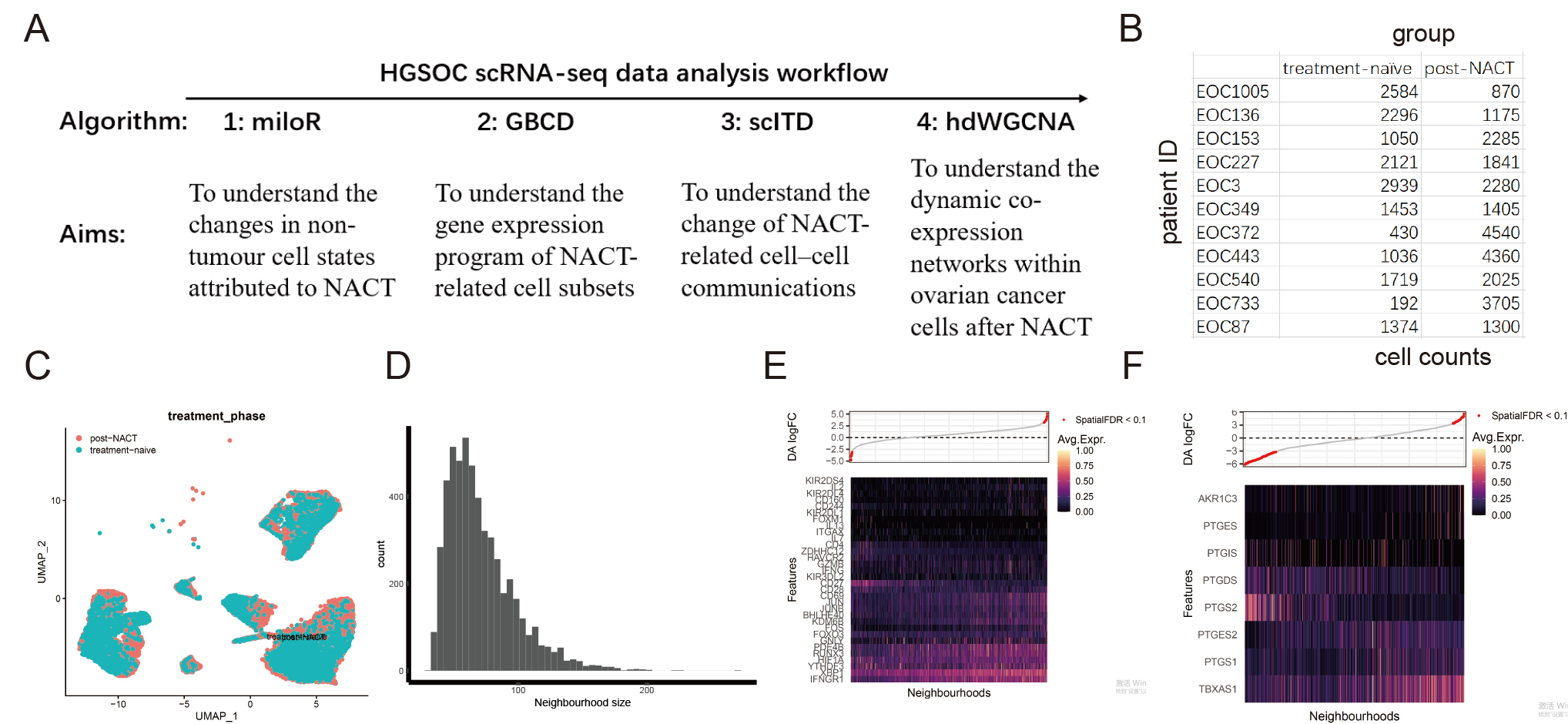

Supplement: Supplementary file 2 — Figure S1 [file 41420_2026_3070_MOESM2_ESM.tif]

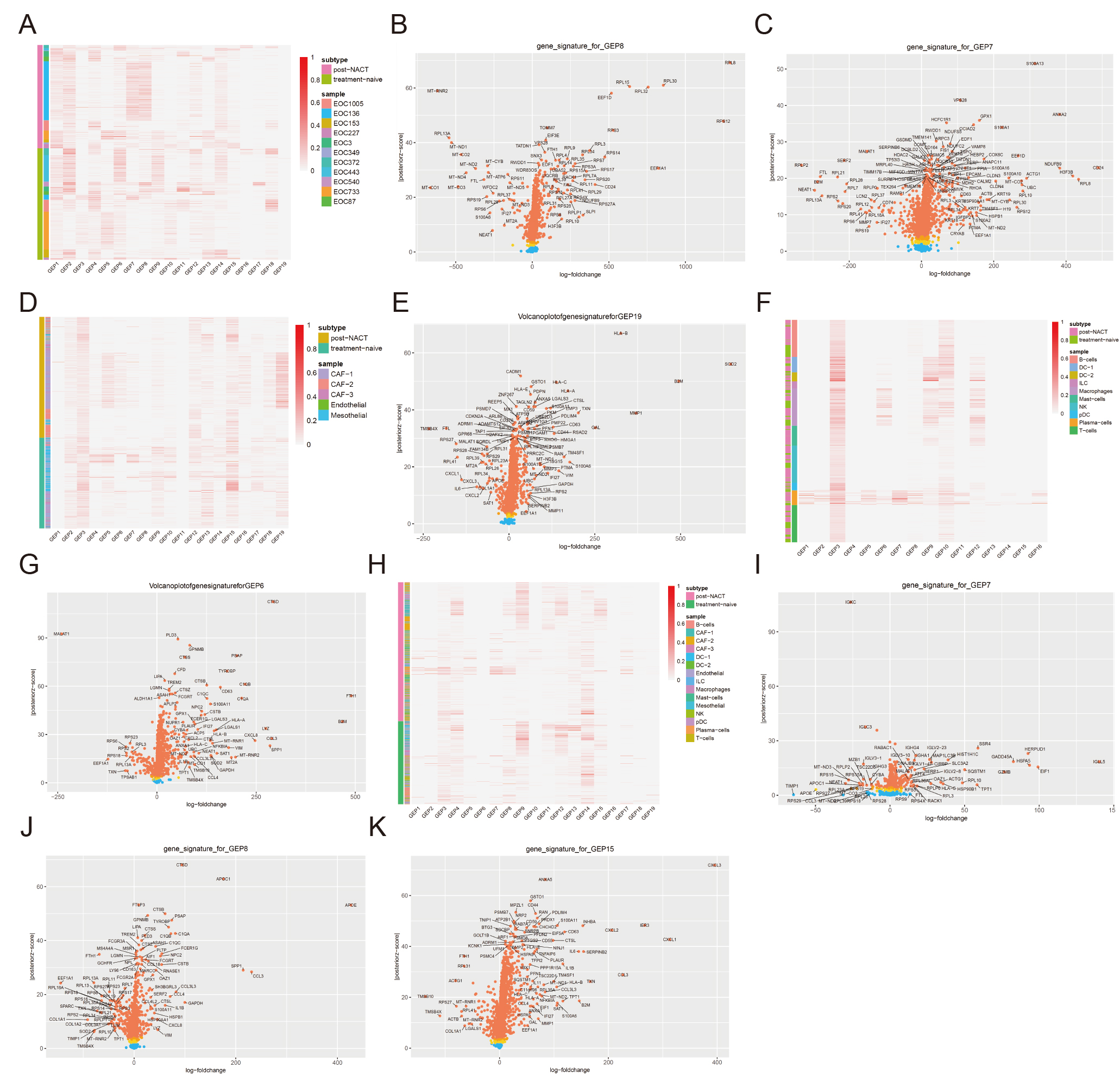

Supplement: Supplementary file 3 — Figure S2 [file 41420_2026_3070_MOESM3_ESM.tif]

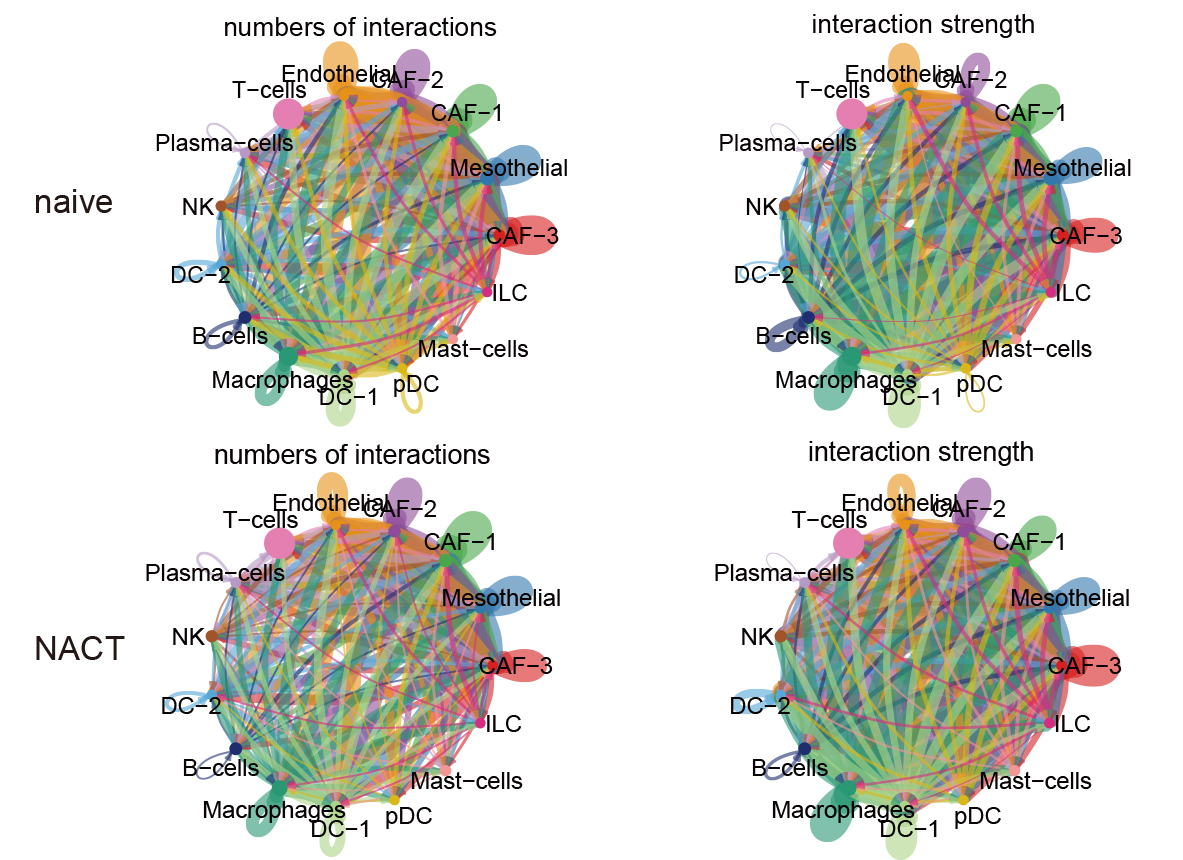

Supplement: Supplementary file 4 — Figure S3 [file 41420_2026_3070_MOESM4_ESM.tif]

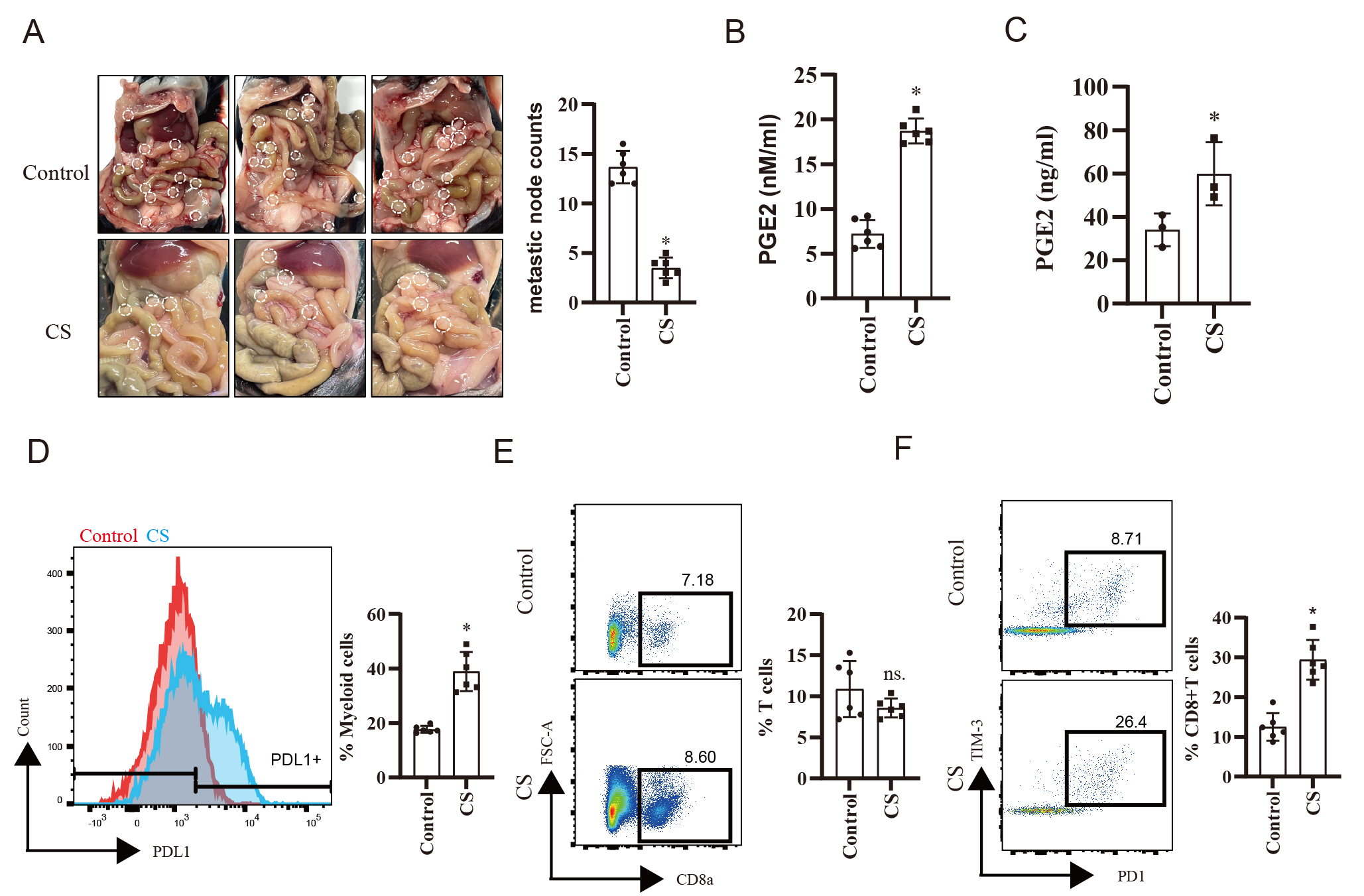

Supplement: Supplementary file 5 — Figure S4 [file 41420_2026_3070_MOESM5_ESM.tif]

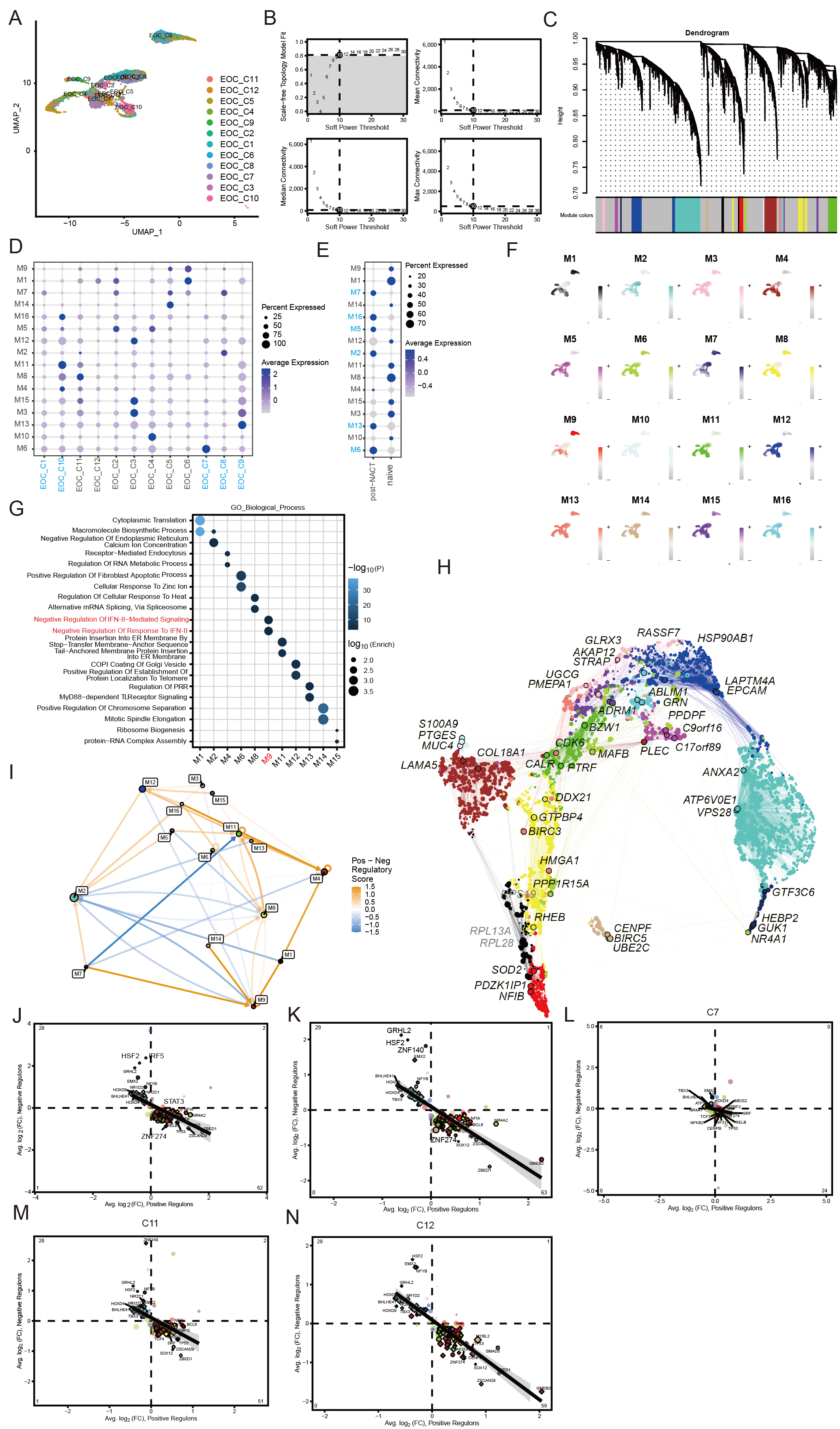

Supplement: Supplementary file 6 — Figure S5 [file 41420_2026_3070_MOESM6_ESM.tif]

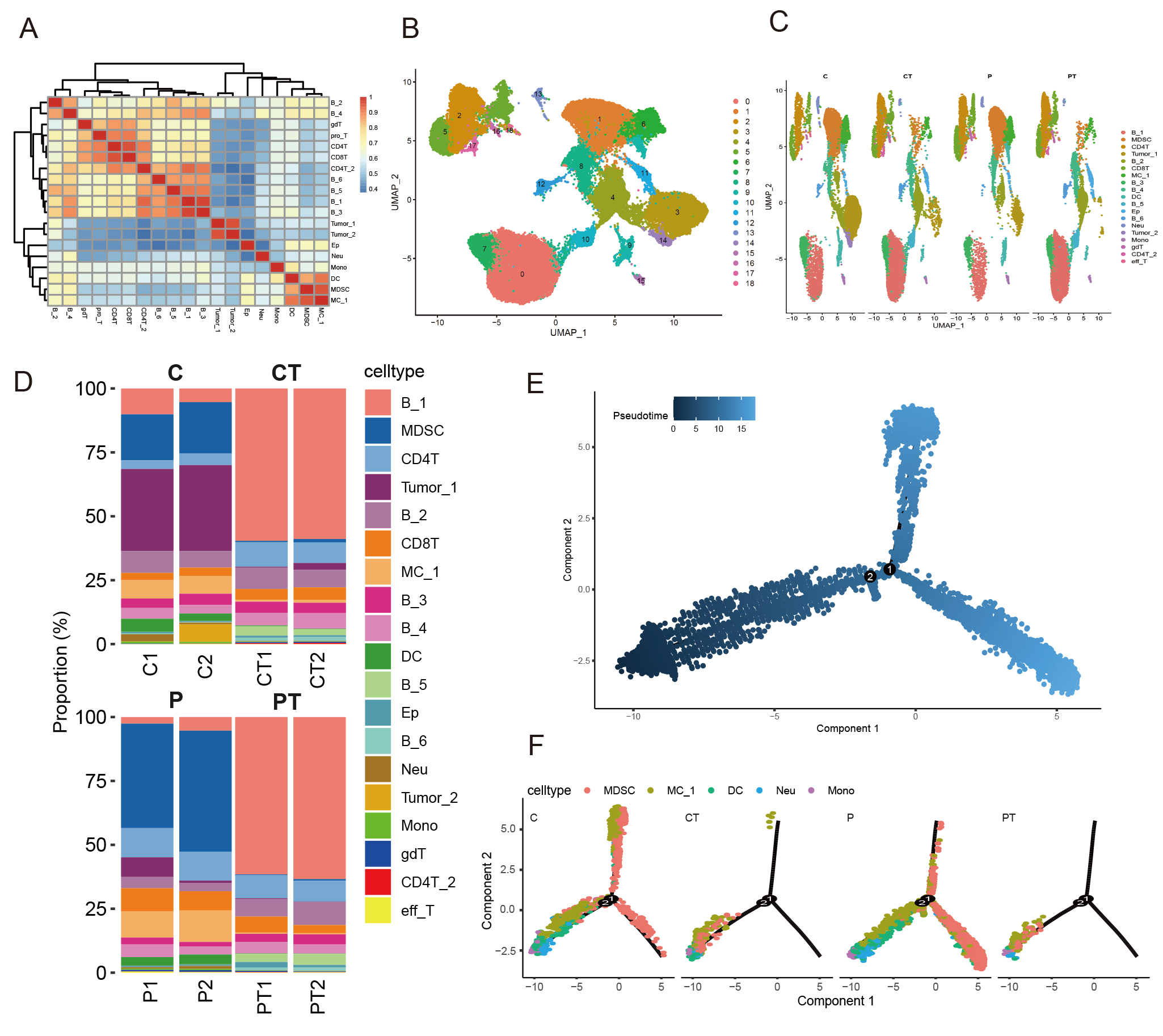

Supplement: Supplementary file 7 — Figure S6 [file 41420_2026_3070_MOESM7_ESM.tif]

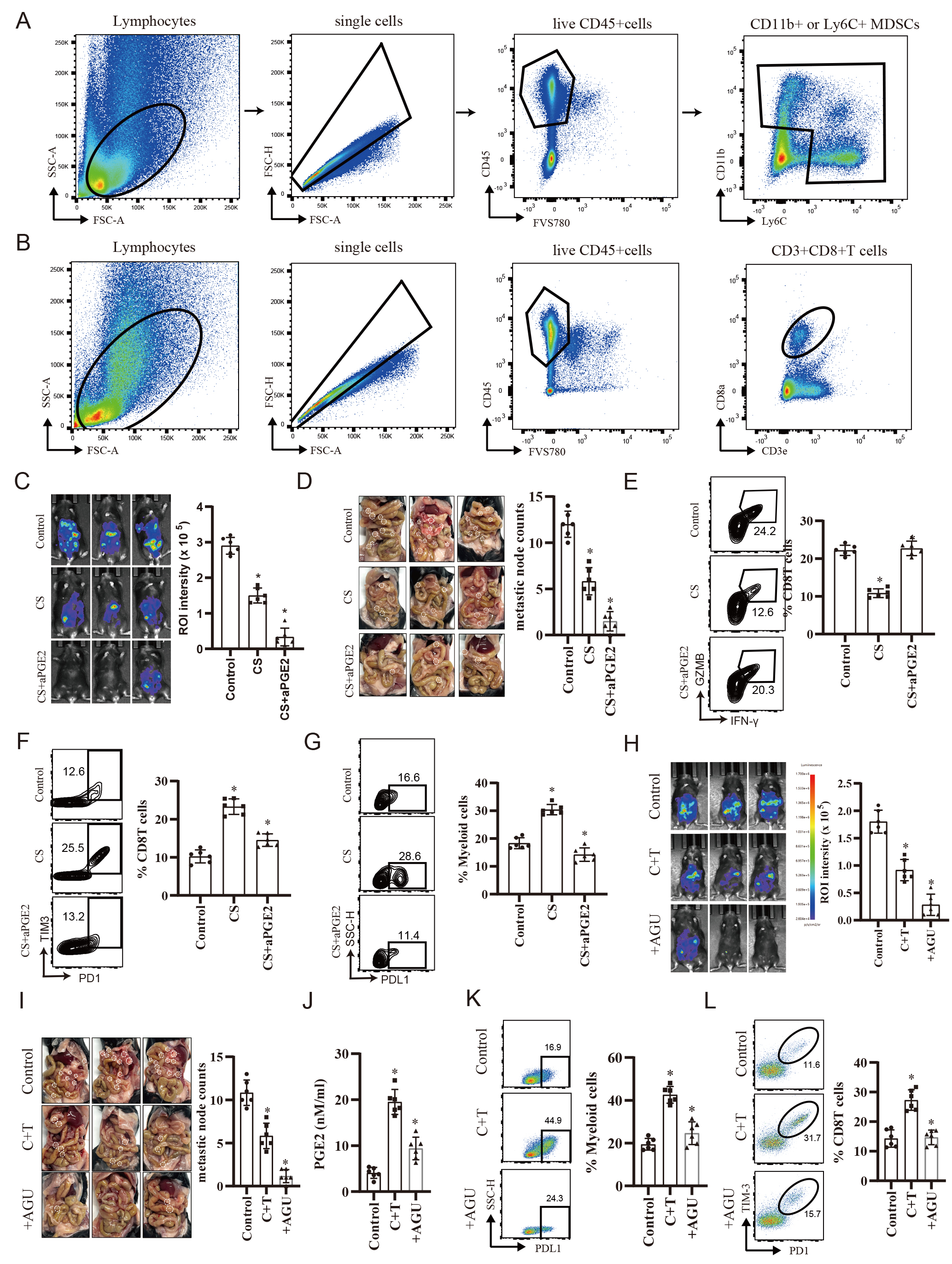

Supplement: Supplementary file 8 — Figure S7 [file 41420_2026_3070_MOESM8_ESM.tif]

**Uncropped western blots:**

Figure 6:


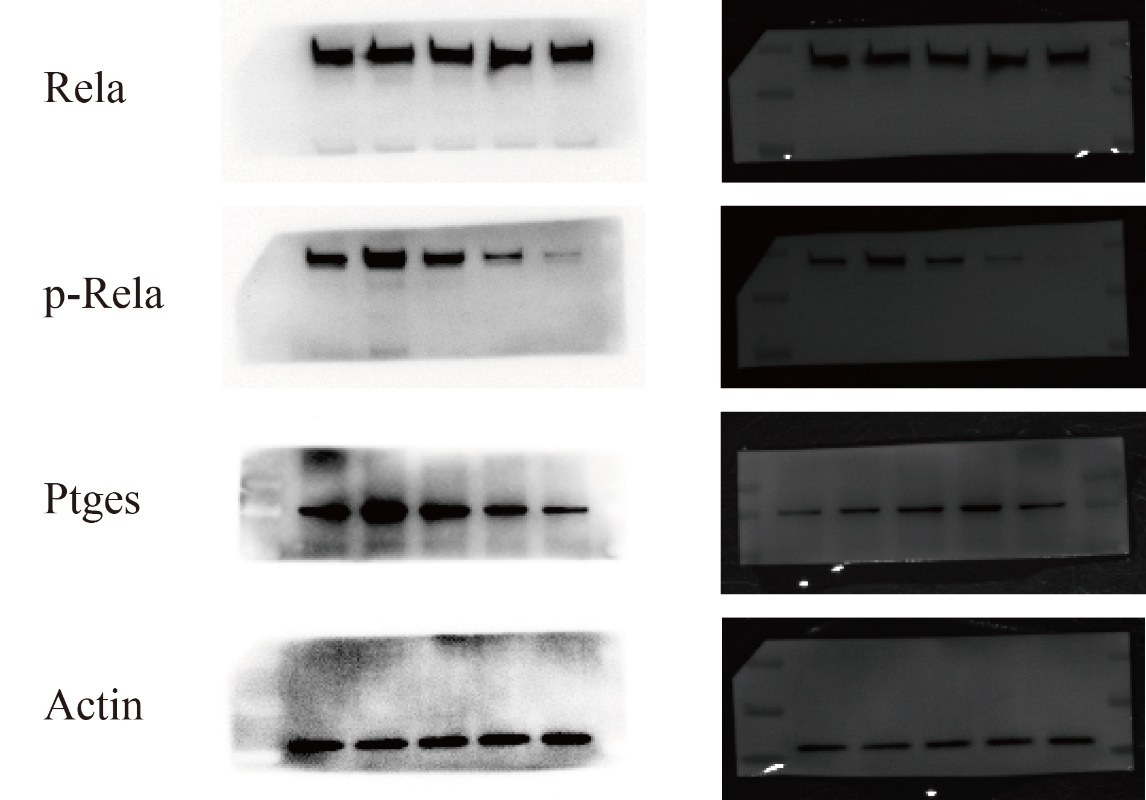

Supplement: Supplementary file 10 — Uncropped western blots [file 41420_2026_3070_MOESM10_ESM.docx]
