# Supplementary material for: Dynamic changes of the immune microenvironment in ovarian cancer following neoadjuvant chemotherapy
Source: Cell Death Discov. 2026 Mar 23;12:130. doi: 10.1038/s41420-026-03070-6 (PMC13039919; doi:10.1038/s41420-026-03070-6)
Supplement: Supplementary file 9 — Supplementary Data [file 41420_2026_3070_MOESM9_ESM.docx]

Supplemental table: Data S1: Patient clinical information.

Supplemental figures:


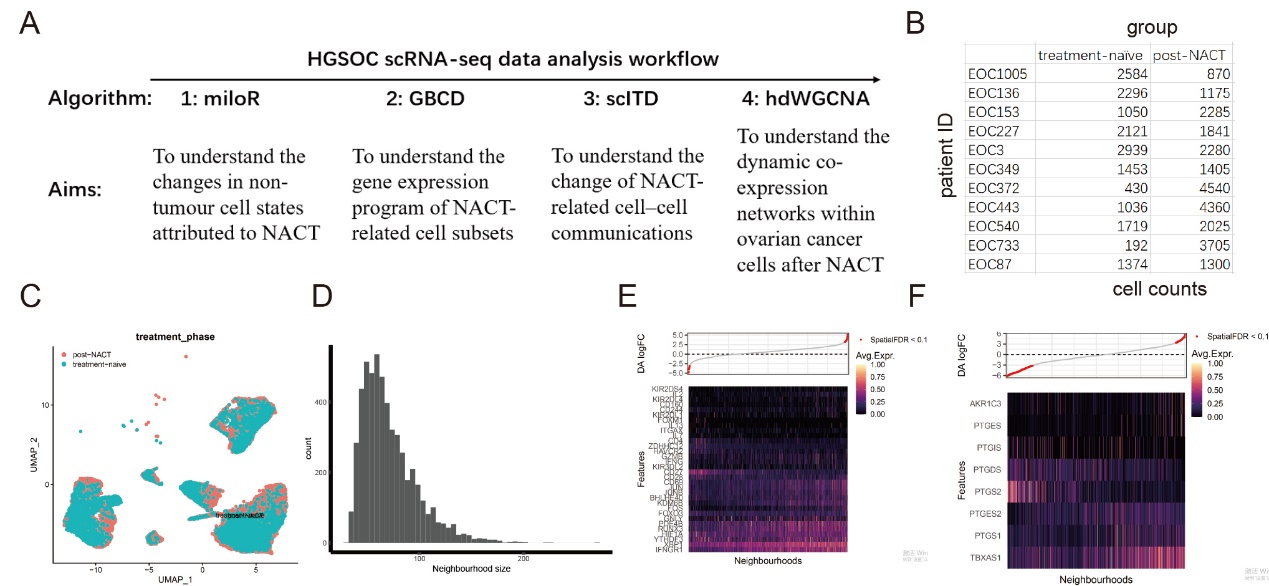


**Figure-S1 MiloR analysis of NACT-related cell subsets and differential genes across pre- and post-treatment.**

A ScRNA-seq data analysis workflow.

B Patient level metadata of the human scRNA-seq dataset (GSE165897).

C Uniform manifold approximation and projection (UMAP) embedding of transcriptomic data across treatment-naïve and post-NACT tumour sections, coloured according to the treatment phase.

D Histogram showing the distribution of neighbourhood sizes.

E Differential abundance (DA) analysis heatmap highlighting the increased expression of NK-related cytotoxic genes (e.g. KIR2DS4 and GZMB) in post-NACT neighbourhoods.

F DA heatmap showing increased expression of prostaglandin pathway genes ( PTGES, PTGDS, and TBXAS1) in post-NACT neighbourhoods.


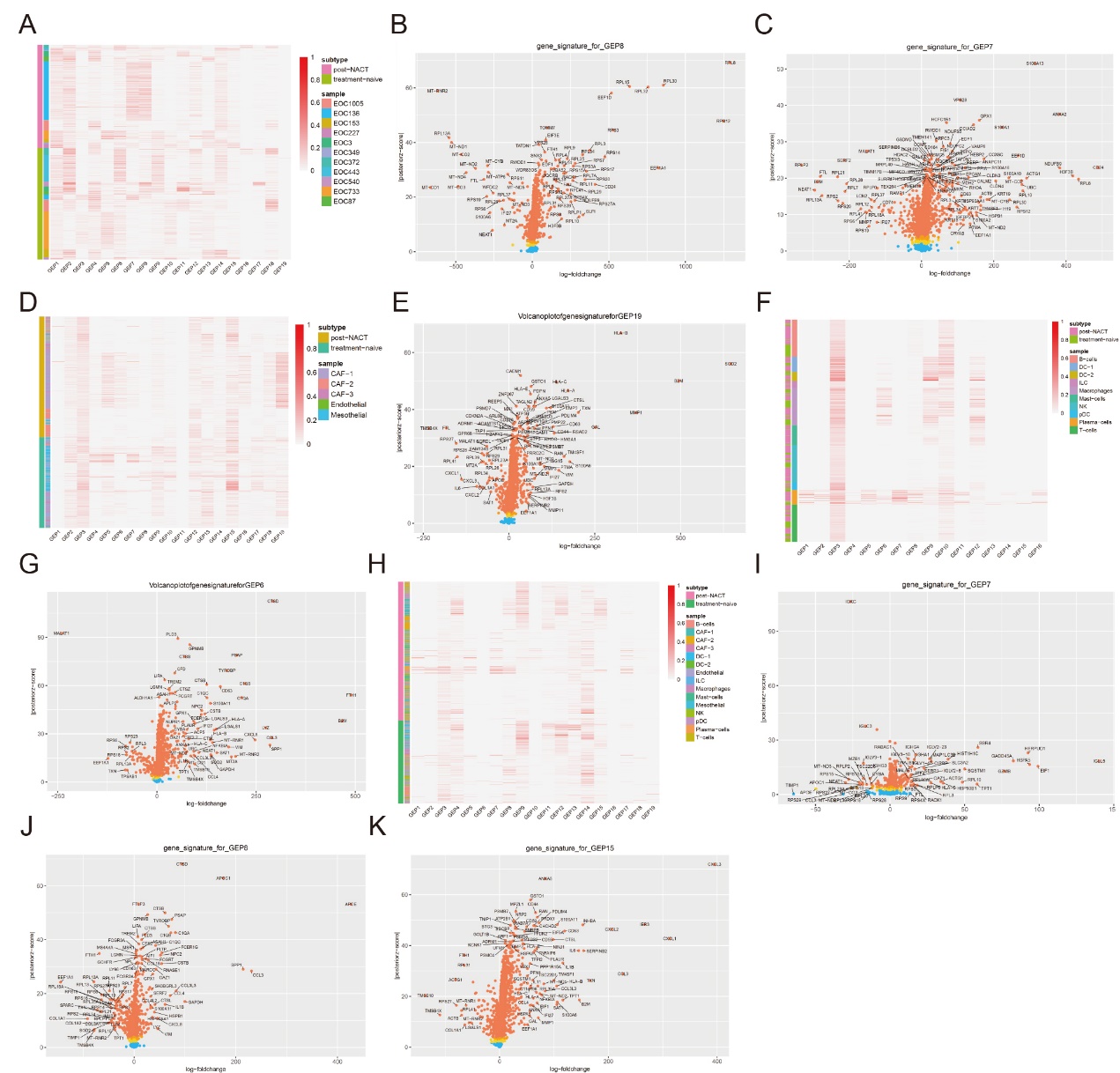


**Figure-S2 Generalised binary covariance decomposition (GBCD) analysis reveals neoadjuvant chemotherapy (NACT)-associated transcriptional changes across cell types in the tumour microenvironment.**

A Cell membership in the 19 gene expression programs (GEPs) estimated by GBCD in the tumour cell subset. Membership values were rescaled separately for each GEP, such that the maximum membership in each GEP was always one.

B-C Volcano plot of differentially expressed genes contributing to the GEP8 (B) or GEP7 (C) signature, highlighting genes significantly upregulated or downregulated after NACT.

D Cell memberships in 19 GEPs estimated by GBCD in the stromal cell subset.

E Volcano plot of differentially expressed genes contributing to the GEP19 signature, highlighting genes significantly upregulated or downregulated after NACT.

F Cell memberships in 19 GEPs estimated by GBCD in the immune cell subset.

G Volcano plot of differentially expressed genes contributing to the GEP6 signature, highlighting genes significantly upregulated or downregulated after NACT.

H Cell membership in the 19 GEPs estimated using GBCD in the non-tumour cell subset.

I-K Volcano plot of differentially expressed genes contributing to the GEP7 (I), GEP8 (J) or GEP15 (K) signature, highlighting genes significantly upregulated or downregulated after NACT.


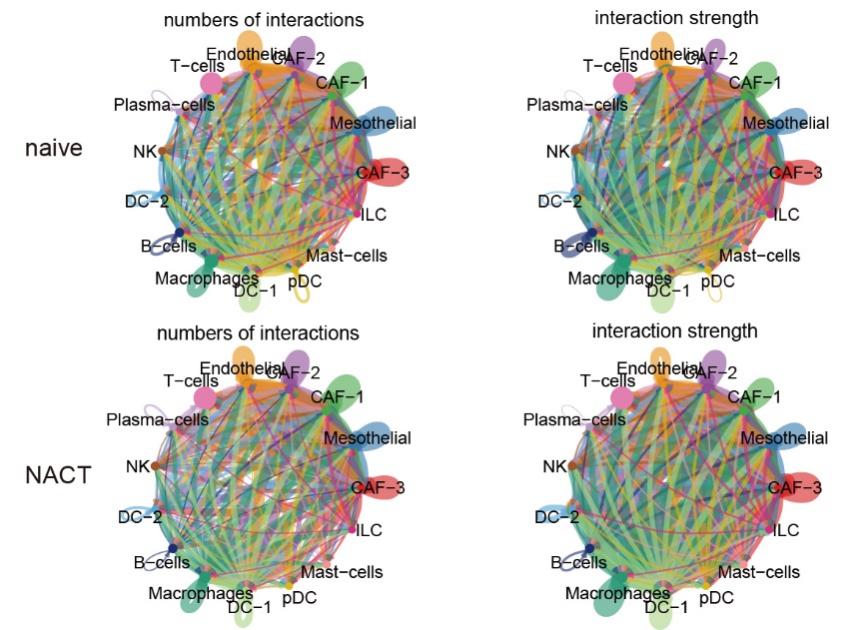


**Figure-S3 Cell–cell communications among non-tumour cells**

Chord diagrams showing the number of strengthened interactions among the 15 major cell types.


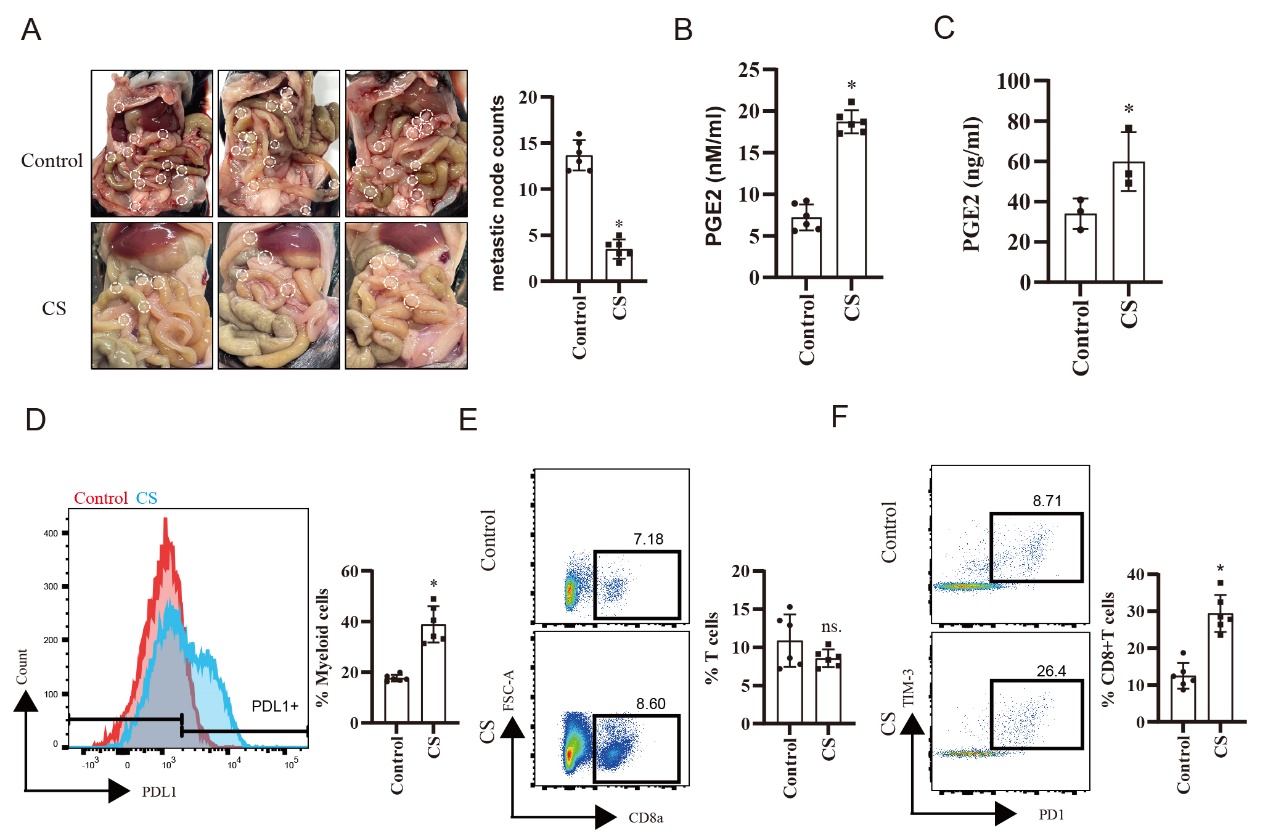


**Figure-S4 Cisplatin impairs tumour progression whereas induces immunosuppressive TME**

A: Images of metastatic nodes in mice peritoneal cavity with or without cisplatin (n = 6, p < 0.05).

B-C ELISA analysis of prostaglandin E_2_ levels in peritoneal irrigation fluid (B) and ID8 cell cultures (C) after chemotherapy (n = 6, p < 0.05).

D Flow cytometry showing an increase in PD-L1^+^ myeloid cells after CS treatment (n = 6, p < 0.05).

E The frequency of CD8^+^T cells in the control and CS groups was determined using flow cytometry (n = 6, p < 0.05).

F TIM-3⁺PD1⁺-exhausted CD8^+^ T cells significantly increased following chemotherapy (n = 6, p < 0.05).


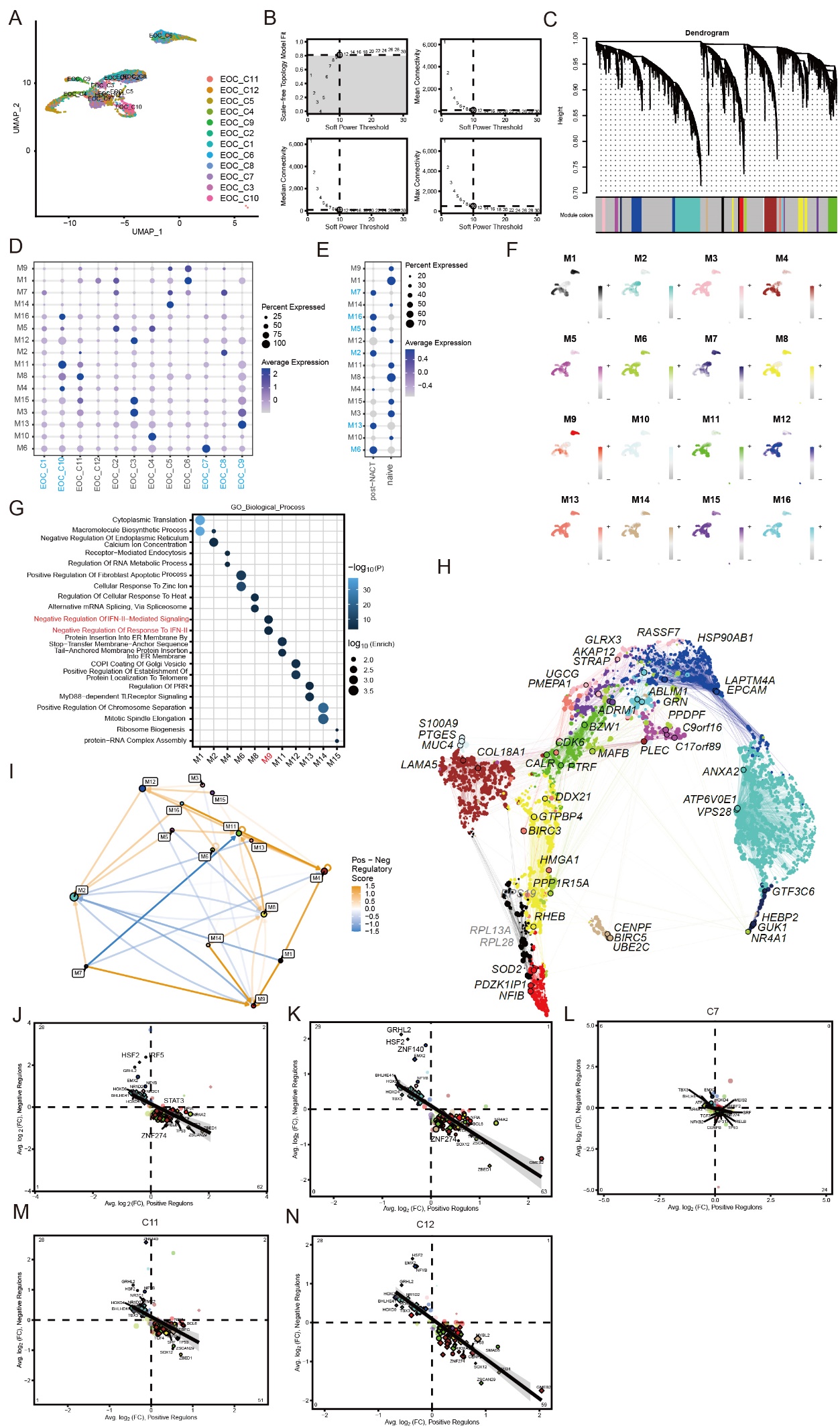


**Figure-S5 Identification of gene modules and transcriptional networks associated with treatment response in epithelial ovarian cancer (EOC).**

A Uniform manifold approximation and projection (UMAP) plot of ovarian cancer cells showing 12 major cell types by manual annotation.

B Soft-thresholding power selection plots based on scale-free topology, mean connectivity, and median connectivity.

C Dendrogram showing the co-expression modules. The grey module should be ignored in all downstream analyses and interpretations.

D Dot plot showing the expression of 16 gene modules (M1–M16) across individual samples.

E Comparison of the average expression levels of modules M1–M16 between the treatment-naïve and post-neoadjuvant chemotherapy (NACT) groups.

F UMAP plots showing the spatial distribution of each gene module (M1–M16) across the ovarian cancer cell subsets.

G Gene ontology (GO) enrichment analysis of the biological processes associated with each Gene module. Notably, M9–M11 were enriched in the negative regulation of type I interferon signalling and response.

H UMAP plot of the EOC co-expression network. Each node represents a single gene, and the edges represent the co-expression links between the genes and module hub genes. The top five hub genes per module are labelled.

I Network plot illustrating the inferred regulatory relationships among gene modules, with edge colours indicating positive (orange) or negative (blue) regulatory scores.

J-K Transcription factor (TF) motif enrichment plots comparing the average log2 fold changes in EOC_C1 (J) or EOC_C8 (K) between positive and negative module-associated regions. TFs, such as STAT3, ZNF274, and HSF2, are identified as potential key regulators.

L-N Differential transcription factor activity analysis of the EOC_C7 (L), EOC_C11 (M), and EOC_C12 (N) modules.


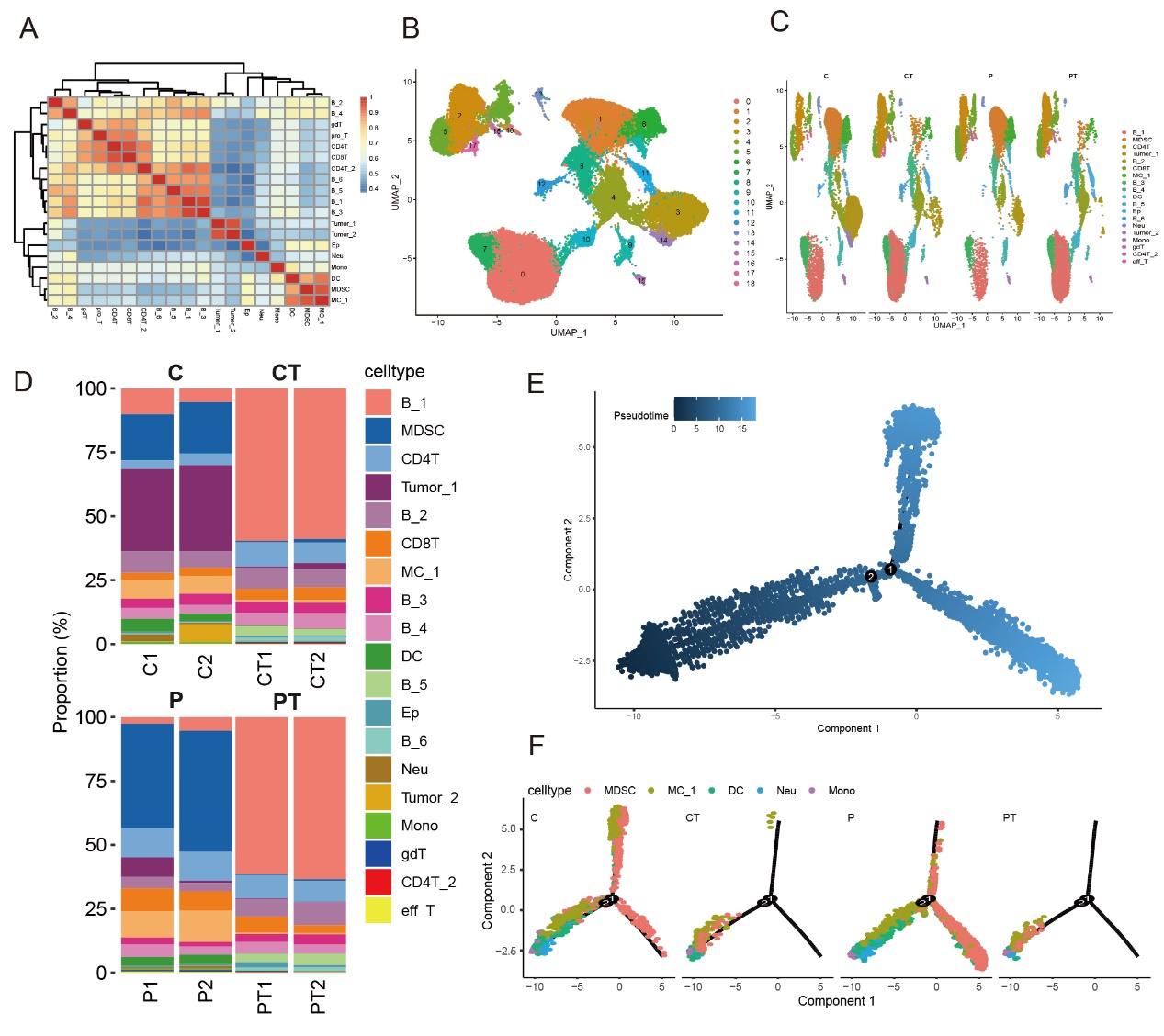


**Figure-S6 Cell types distribution and pseudo-time analysis of myeloid cells**

A Cell similarity analysis among all cell subsets.

B Uniform manifold approximation and projection (UMAP) plot showing 19 major cell lineages across the four groups.

C UMAP plot showing 19 major cell lineages grouped by sample and treatment.

D Bar charts showing the ratios of 19 major cell lineages across the four groups (C, CT, P, and PT).

E-F Monocle3 pseudo-time trajectory plots show dynamic lineage progression and treatment-induced shifts in the immune cell state, with colour coding by pseudo-time (D) or cell type (E).


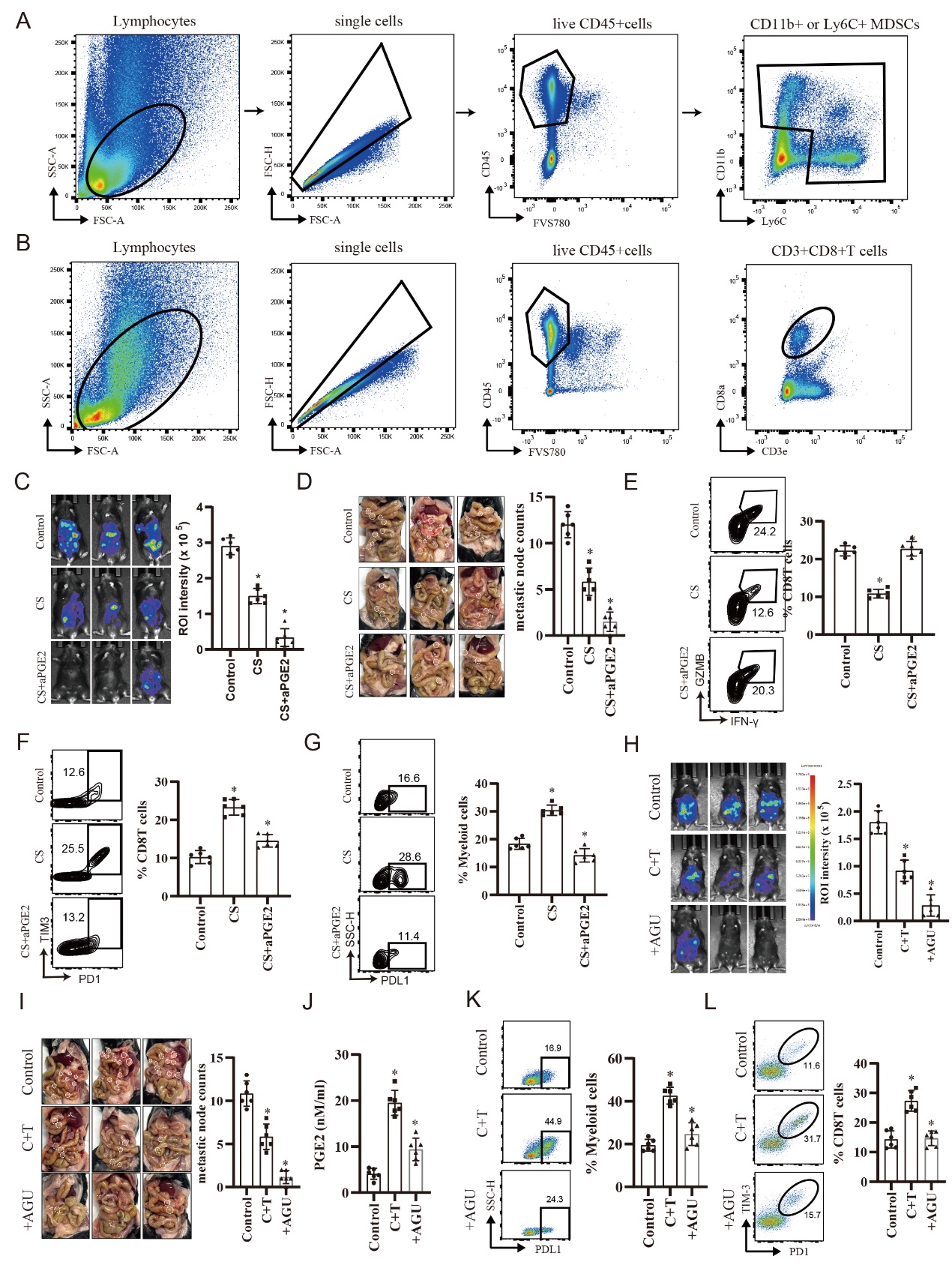


**Figure-S7 Flow cytometry gating strategy and therapeutic effects of AGU treatment.**

A-B Gating strategy for the identification of myeloid-derived suppressor cells (MDSCs) (A) and CD8^+^ T-cells (B).

C-D Female mice were intraperitoneally-injected with luc-ID8 (stably expressing the luciferase gene promoter) cells for two weeks. Subsequently, cisplatin or cisplatin plus aPGE2 was administered twice per week. In vivo imaging was performed on 4th week. The right panel shows the results. The representative pictures are shown (C) (n = 6, p < 0.05); Representative images of metastatic nodules in the peritoneal cavity from each treatment group, with bar graph quantifying the number of visible metastases. Combination treatment further reduced the metastatic spread (n = 6, p < 0.05).

E-G Flow cytometry showing reduced PD-L1^+^ myeloid cells, decreased CD8^+^ T-cell exhaustion and increased cytotoxicity of CD8^+^T in aPGE2-treated mice (n = 6, p < 0.05).
H-I Female mice were intraperitoneally-injected with luc-ID8 cells (stably expressing the luciferase gene promoter) for two weeks. Subsequently, cisplatin/paclitaxel (C+T) or C+T+AGU therapy was administered twice weekly. In vivo imaging was performed on 4^th^ week. The right-hand panel shows the results. The representative pictures are shown (H) (n = 6, p < 0.05); Representative images of metastatic nodules in the peritoneal cavity from each treatment group, with bar graph quantifying the number of visible metastases. Combination treatment further reduced the metastatic spread (n = 6, p < 0.05).
J Prostaglandin E_2_ concentration is reduced after AGU therapy compared to that in the C+T group (n = 6, p < 0.05).
K-L Flow cytometry showing reduced PD-L1^+^ myeloid cells and decreased CD8^+^ T-cell exhaustion in AGU-treated mice (n = 6, p < 0.05).
